# Effects of Endurance Exercise and Vitamin D Supplementation on Insulin Resistance and Plasma Lipidome in Middle-Aged Adults with Type 2 Diabetes

**DOI:** 10.3390/nu15133027

**Published:** 2023-07-03

**Authors:** Xiaomin Sun, Tao Yan, Zhongying Li, Sirui Zhou, Wen Peng, Wei Cui, Jing Xu, Zhen-Bo Cao, Lin Shi, Youfa Wang

**Affiliations:** 1 Global Health Institute, School of Public Health, Xi’an Jiaotong University Health Science Center, Xi’an 710061, China; gzhtxiaomin@163.com (X.S.); lzying1215@163.com (Z.L.); 2 School of Food Engineering and Nutritional Science, Shaanxi Normal University, Xi’an 710119, China; yantao@snnu.edu.cn; 3 Department of Administrative Management, First Affiliated Hospital, Xi’an Jiaotong University, Xi’an 710061, China; zhousirui1108@sina.com; 4 Nutrition and Health Promotion Center, Department of Public Health, Medical College, Qinghai University, Xining 810061, China; wen.peng2014@foxmail.com; 5 Department of Geriatric Endocrinology, First Affiliated Hospital, Xi’an Jiaotong University, Xi’an 710061, China; doctorweiwei.cui@126.com; 6 Department of Endocrinology, Second Affiliated Hospital, Xi’an Jiaotong University, Xi’an 710061, China; xujingjdey85@163.com; 7 School of Kinesiology, Shanghai University of Sport, 399 Chang Hai Road, Shanghai 200438, China

**Keywords:** exercise, vitamin D, lipidomic profile, diabetes, insulin resistance

## Abstract

(1) Background: We investigated the effects of a 12-week exercise intervention with or without vitamin D supplementation on insulin resistance and the plasma lipidome of participants with type 2 diabetes. We further explored whether the effects of the intervention on glycemic parameters could be associated with the baseline lipidome. (2) Methods: Sixty-one participants were randomly allocated to control (Con), exercise (EX), vitamin D (VD), and EX + VD groups. Multiple glycemic and anthropometric parameters were evaluated before and after intervention. The homeostasis model assessment of insulin resistance (HOMA-IR) was the primary outcome. The plasma lipidome was analyzed before, after, and at an additional 12-week follow-up. Machine learning was applied to establish prediction models for responsiveness of glycemic control. (3) Results: Our interventions failed to improve the HOMA-IR index while fasting glucose was reduced in the EX + VD group (change%, −11.9%; effect size, 0.65; *p* < 0.05). Both EX and VD interventions altered the plasma lipidome, with EX + VD intervention considerably affecting levels of lyso-phosphatidylcholines and triglycerols containing long-chain unsaturated fatty acids. Such effects could last until 12 weeks after intervention. Notably, there was high inter-individual variability in glycemic parameters including HOMA-IR in response to the interventions, which could be predicted with great accuracy using an optimal panel of baseline lipid predictors alone or in combination with clinical indices, as assessed by an area under the receiver operating characteristic curve value of over 0.9. (4) Conclusions: Although substantial alterations were observed in the plasma lipidome related to glycemic control, our intervention failed to improve HOMA-IR scores, which may have been predominately due to the large inter-individual variability in responses. Basal plasma lipid levels could potentially predict an individual’s response to intervention, highlighting the necessity of personalized nutrition.

## 1. Introduction

Due to rapid social and economic development during the past three decades, China is facing a growing threat from non-communicable chronic diseases (NCDs), with diabetes considered to be one of the most common NCDs in China and several other countries [1]. The most recent nationally representative surveys revealed that diabetes prevalence among Chinese adults had increased from 10.9% in 2013 to 12.4% in 2018 [2]. 

Endurance exercise has been recommended as an effective intervention for the prevention and treatment of type 2 diabetes mellitus (T2DM) by several associations, such as the American College of Sports Medicine and the American Diabetes Association. Moreover [3,4], moderate endurance exercise with a target maximum heart rate (60–85%, submaximal exercise) is also recommended by the National Fitness Guidance for China and Chinese Diabetes Society for T2DM prevention and control [5,6]. However, the findings of randomized controlled trials (RCTs) regarding the impacts of exercise on the homeostasis model assessment of insulin resistance (HOMA-IR) index and glycated hemoglobin (HbA1c) levels remain inconsistent [7,8]. Considering the substantial impact of vitamin D on the physiology of T2DM (i.e., directly upregulating the AMP-activated protein kinase [AMPK]-glucose transportor-4 [GLUT-4] signaling pathway) [9], co-exposure to vitamin D has been shown to provide additional benefits for physical functioning in older adults [10]. Our previous work showed that higher levels of cardiorespiratory fitness and serum 25(OH)D concentrations due to exercise training were associated with lower HOMA-IR scores than exercise alone in middle-aged and elderly men [11]. However, few RCTs have assessed the potential synergistic effects of exercise combined with vitamin D intervention on glycemic control and the underlying mechanisms in participants with T2DM. 

Dyslipidemia is a well-known risk factor for T2DM, which is clinically characterized by abnormal regulation of total cholesterol and triglycerides, low-density lipoprotein cholesterol (LDL-C), and high-density lipoprotein cholesterol (HDL-C) [12,13,14]. It reflects remarkable perturbations in several circulating molecular lipid species, including glycerolipids, phospholipids, sphingolipids, and ceramides (Cers), which are involved in various biological functions in relation to glucose homeostasis [15,16,17]. Moreover, plasma levels of triglycerols (TGs) and phosphatidylcholines (PCs) have been identified as potential predictive and diagnostic biomarkers of T2DM using high-throughput lipidomics in cohorts [15,16]. Notably, an increasing number of studies have reported inter-individual differences in response to diet or exercise treatments [18,19], and associations have been shown to exist between baseline metabolomics phenotypes and intervention-induced improvements in metabolic health outcomes [20,21]. However, to our knowledge, no studies have investigated the detailed influence of exercise combined with vitamin D intervention on molecular lipid species in relation to glucose homeostasis in participants with T2DM and their predictive capacity at baseline. Such information is of importance in identifying targets for T2DM management using lifestyle interventions. 

The primary aim of the present study was to investigate the effects of a 12-week endurance exercise training program with or without vitamin D supplementation on glycemic control (i.e., HOMA-IR and HbA1c), and its related plasma lipid species in participants with T2DM using lipidomics. We also assessed the post-intervention effects on lipids after an additional 12-week follow-up. In addition, our investigation was extended to investigate whether the basal plasma lipidome could predict the individual’s response to interventions in terms of glycemic control improvement. We hypothesized that exercise combined with vitamin D intervention would have synergistic effects on glycemic control and specific lipids, and the changes in lipids would be associated with glycemic improvement. Furthermore, basal specific lipids would have greater predictive potential for changes in glycemic control than traditional clinical indices.

## 2. Methods

### 2.1. Study Design and Population

The study was a randomized controlled trial with a 12-week intervention and additional 12-week follow-up period assessing the impact of vitamin D and exercise intervention on glycemic improvement in participants diagnosed with T2DM without insulin treatment in Xi’an, China (34° N latitude). Participants were excluded if they had (a) past insulin therapy or a plan to change hypoglycemic drugs during the trial; (b) ≥400 IU/day vitamin D supplementation for a month or regular exercise training; (c) acute infection; stress; heart, liver, and renal insufficiency; osteoporosis; or fracture; (d) a history of sunbathing at the beach, at a swimming pool, or anywhere else in the last six months; or (e) use of metal implants or other factors that could influence dual-energy X-ray absorptiometry (DXA) testing. 

**Figure 1:**
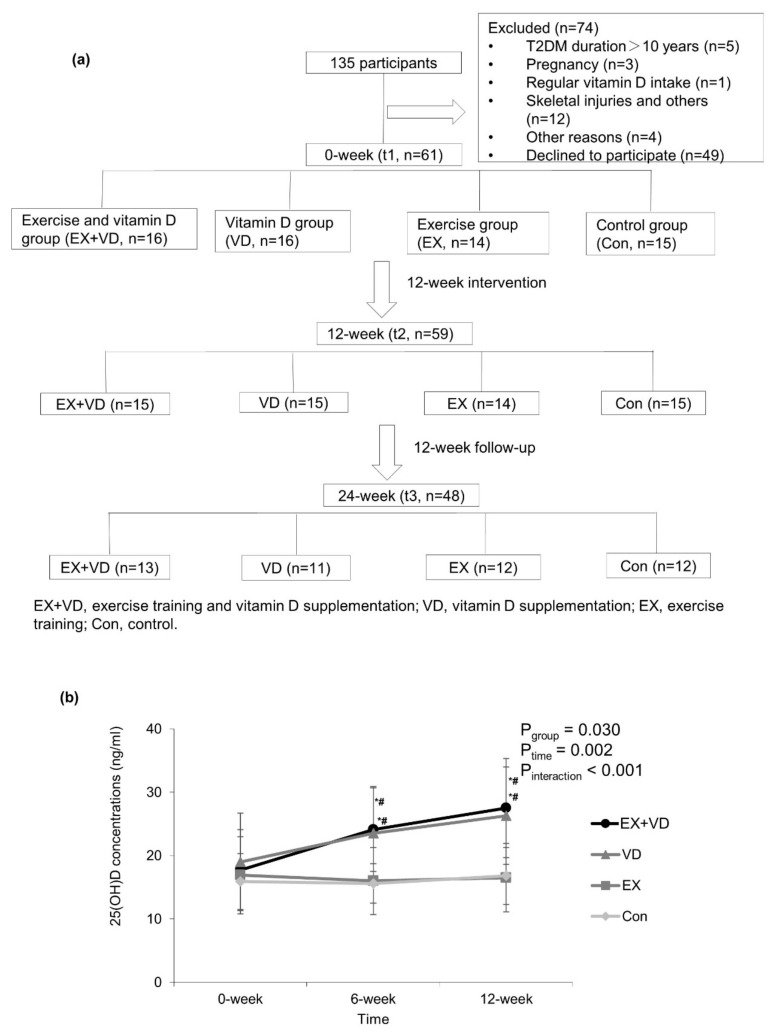
Flow diagram of participants (**a**) and changes in serum 25(OH)D concentrations during 12-week interventions (**b**). Values are expressed as mean ± standard deviation. Two way repeated-measures ANOVA was used to determine the effect of vitamin D and exercise on serum 25(OH)D changes. EX + VD, exercise training and vitamin D supplementation group; VD, vitamin D supplementation group; EX, exercise training group; Con, control group. EX + VD, *n* = 15; VD, *n* = 15; EX, *n* = 14; Con, *n* = 15. *, *p* < 0.05 vs. baseline within group; #, *p* < 0.05 vs. exercise or control group at the same time point.

Participants were randomly allocated into four groups using a computer-generated random number sequence: exercise + vitamin D group (EX + VD: *n* = 16), exercise group (EX: *n* = 14), vitamin D group (VD: *n* = 16), and placebo control group (Con: *n* = 15). The details of participants’ inclusion and exclusion are shown in Figure 1a.

Based on our previous study examining the association of vitamin D intervention with HOMA-IR [22], the sample size required for each group was estimated to be 12 with a power of 85%, and an effect size of 0.26 was assumed. To account for 20% loss to follow-up, 60 participants were recruited. Power calculations were performed using G*Power software version 3.1.9.2 [23]. 

All of the procedures were reviewed and approved by the Ethics Committee of Xi’an Jiaotong University Health Science Center. The study was conducted in accordance with the Declaration of Helsinki and registered in the Chinese Clinical Trial System (No. ChiCTR1800015383).

### 2.2. Intervention

#### 2.2.1. Vitamin D Intervention

Participants in the EX + VD and VD groups took a daily vitamin D_3_ supplement of 1000 IU (Nature Made, Otsuka Pharmaceutical Co, Ltd., Osaka, Japan), while those in the EX and Con groups received a placebo tablet identical to the vitamin D_3_ supplement in appearance, shape, and color. The placebo tablets contained only starch, cellulose, and magnesium stearate. All tablets were presented in identical bottles and were sent or hand-delivered to the homes of participants monthly, and participants were asked to report remotely each week. 

#### 2.2.2. Exercise Intervention

Participants in the EX and EX + VD groups participated in a supervised 1-h progressively increasing endurance cycling exercise at 65–80% of maximal heart rate (HRmax), 2 to 3 times/week, for 12 weeks at a gym. HRmax was predicted using the following formula: HRmax = (220 − age) [24]. The detailed exercise protocol for each week was provided elsewhere [25] and is attached as Supplementary Table S1. The exercise training was supervised by a qualified trainer who was knowledgeable of the study protocol and procedures. The exercise intensity, evaluated by HR, and rating of perceived exertion (RPE) was monitored during exercise and the trainer ensured that it matched the prescribed values as indicated in Supplementary Table S1. A polar monitor was used to track heart rate during exercise, and compliance with each protocol was recorded. If the actual HRs and RPEs were below or above the prescribed values, we adjusted the exercise load until the participants fell within the prescribed intensity range. Participants warmed up during the first 5 min on a treadmill at 50–60% of HRmax and subsequently followed the exercise protocol assigned to them, with a 5 min recovery exercise period at 40–50% of HRmax, which comprised walking and stretching exercises. Participants in the VD and Con groups were asked to maintain their previous lifestyle habits during the trial. 

### 2.3. Data Collection

#### 2.3.1. Anthropometric Measurements and Questionnaires

Body mass index (BMI) was calculated as body mass in kilograms divided by height in meters squared (kg/m^2^). Body fat mass (kg), body muscle mass (kg), and body fat percentages (%) were estimated using DXA (Hologic QDR-4500, DXA Scanner, Hologic Inc., Newark, DE, USA) by a recognized technologist. The average systolic blood pressure (SBP) and diastolic blood pressure (DBP) of ankle and arm were measured using a non-invasive vascular screening device (BP-203RPE II device; Omron Healthcare, Kyoto, Japan).

Physical activity information was obtained using the long form of the International Physical Activity Questionnaire (IPAQ), except for when participants were attending the exercise program. Metabolic equivalent task (MET) values in minutes/week were calculated [26]. The mean weekly sunlight exposure score was calculated from sun exposure time and exposed skin area. Additional details are provided elsewhere [27]. The above indicators were determined at t1 and t2.

#### 2.3.2. Glycemic Control Indicators

Venous fasting blood samples were collected at baseline (t1), 12 weeks (t2), and the 12-week follow-up (t3) after a 12-h overnight fast in Venoject-II AutoSep tubes with serum or NaF for plasma. Tubes with EDTA were used to determine blood glycosylated hemoglobin (HbA1c). Serum and plasma samples were transferred to separate tubes and frozen immediately at −80 °C. Fasting samples were used to measure the levels of plasma glucose, serum insulin, serum triglyceride, serum total cholesterol (TC), serum low-density lipoprotein cholesterol (LDL-C), serum high-density lipoprotein cholesterol (HDL-C), serum apolipoprotein (Apo)A-1, serum ApoB, and serum liver and renal function indicators (e.g., uric acid, creatinine, and γ-GT) by KingMed Diagnostics Inc. (Xi’an, China). At t1 and t2, a standard 75 g oral glucose tolerance test (OGTT) was also performed between 08:30 and 11:00, and plasma glucose and serum insulin levels were determined at 0, 30, 60, 90, and 120 min. Serum samples obtained at 6 and 12 weeks during the interventions were subjected to 25(OH)D analysis using electrochemiluminescence immunoassays (Roche Diagnostics GmbH, Mannheim, Germany). The intra- and inter-assay variation coefficients for 25(OH)D were 1.96% and 3.78%, respectively. 

The HOMA-IR index was the primary endpoint and calculated as follows: HOMA-IR = G_0_ × I_0_/22.5 [28]. Insulin sensitivity was estimated using the Matsuda index during the OGTT and was calculated as follows: 1000/square root of ([G_0_ × I_0_]) × (mean OGTT glucose concentration × mean OGTT insulin concentration) [29]. The increments in the area under the curve (AUC) of glucose and insulin during the complete 120-min period of the OGTT (all 5 points) were calculated using the trapezoid rule to assess glucose tolerance and insulin secretion [30].

#### 2.3.3. Plasma Lipidomic Profiling

Venous plasma samples collected at three time points (t1–t3) were used for lipidomics analysis using ultra-high-performance liquid chromatography–mass spectrometry (UPLC-MS/MS). The order of the plasma samples was randomized prior to extraction and instrumental analyses to minimize the influence of signal fluctuations. Pooled samples were used as quality controls throughout the analysis to verify the reproducibility. Details of instrumental analysis and data processing procedures including lipid extraction, peak detection, and lipid annotation are presented in Supplementary Table S2. 

In brief, 100 μL of plasma sample was properly mixed with 800 μL cold methyl tert-butyl ether and then mixed with 240 μL methanol. The mixture was vortexed for 30 s, sonicated at 4 °C for 20 min, and then centrifuged at 14,000× *g* for 15 min at 10 °C. The upper organic layer containing lipids was then dried in a vacuum centrifuge for instrumental analyses. The lipid extracts of the plasma samples were analyzed using a UPLC instrument (LC30a, Nexera, Quebec, QC, Canada) equipped with a Waters ACQUITY Premier CSH C18 column (1.7 µm, 2.1 × 100 mm). Mass detection was performed using a Q Exactive mass spectrometer (Thermo Scientific™, Waltham, MA, USA) equipped with jet stream electrospray ionization. Data were acquired in both positive and negative ion modes, respectively. Quality control measures were applied throughout the dataset, including the inclusion of blanks, pure standard samples, extracted standard samples, and pooled plasma samples from this study. LipidSearch 4.0 software was used for peak detection and lipid annotation. 

### 2.4. Statistical Analysis

All statistical analyses were conducted using R 4.0.4 or SPSS 24.0. 

Descriptive statistics were calculated using mean ± standard deviation for continuous variables, *n* (%) for categorical variables, or 95% confidence interval (CI), unless otherwise stated. The Kolmogorov–Smirnov test was performed to assess data distribution normality, and non-normally distributed data were normalized by logarithmic transformation. Differences between multiple groups were assessed by ANOVA for continuous variables or chi-squared tests for categorical variables. Paired Student’s test was used to assess the within-group difference. A 2 × 2 factorial design was used to estimate the main or interactive effects of vitamin D and exercise intervention on glycemic control indicators with a Bonferroni post hoc test. An intention-to-treat analysis was performed for all data using SPSS version 22.0 software (SPSS, Inc., Chicago, IL, USA). Statistical significance was set at *p* < 0.05.

Comprehensive changes in lipid profiles in response to different interventions were captured using the fuzzy c-means clustering algorithm (R package “Mfuzz”), followed by a paired Student’s test to assess differences between t1 and t2 in each treatment group. Data were auto-scaled prior to analysis. Specific attention was paid to lipids that were not altered in the Con group but showed pronounced changes between t1 and t2 in the VD, EX, or EX + VD groups (*p* < 0.05 and/or abundance at t2 increased or decreased by 20% compared to that at t1). 

To identify lipids with similar physiological and molecular characteristics that were altered by EX, VD, or EX + VD intervention, a co-expression network of the plasma lipids was constructed using weighted gene correlation network analysis (R package “WGCNA”). A signed hybrid network and power of 8 were used for the scale-free topology criterion (model fitting index R^2^ > 0.8). Lipids were hierarchically clustered using a dynamic tree-cutting algorithm with a minimum module size of 10, resulting in lipid patterns with high intra-correlations (i.e., the module eigenlipid, ME). Partial Pearson correlations between WGCNA-derived lipid patterns and clinical indices (e.g., LDL-C, HDL-C, Total-C, triglycerides, ALT, γ-GT, AST, fasting glucose, fasting insulin, 25(OH)D, BMI, body fat (%), body fat, body muscle mass, blood pressure, physical activity, sun exposure score) altered by VD, EX, or EX + VD intervention were computed, adjusting for age, gender, and intervention. 

Plasma lipids and/or measured clinical indices at baseline were subjected to a random forest algorithm incorporated into a repeated double cross-validation framework with unbiased variable selection (R package “MUVR”) to identify optimal panels of markers that could discriminate responders (i.e., participants who benefitted from the interventions and showed within-individual differences in glycemic control indicators before and after intervention) from non-responders (i.e., participants who underwent interventions but did not exhibit improved glycemic control indicators). Prediction accuracy was determined by misclassification rate % and area under the receiver operating characteristic curve (AROC, 95% CI).

## 3. Results

### 3.1. Baseline Characteristics of Participants

One participant in the VD group and another in the EX + VD group withdrew from the study due to personal reasons during the intervention. Additionally, 11 participants were lost to follow-up at t3. See more details in Figure 1a. Of the 29 participants who participated in exercise training, 20 exercised at least twice a week, and 5 exercised 1–2 times a week. The frequency of attendance at exercise sessions did not differ between the EX and EX + VD groups. No trial-related adverse events were reported during the intervention. As shown in Table 1, the average age of 59 participants was 50.1 ± 7.3 years, with a mean BMI of 25.9 ± 3.6 kg/m^2^ at baseline; of them, 44 (71.2%) were men. No differences were observed in basal BMI and glycemic indicators among the groups. 

### 3.2. Effects of Exercise and Vitamin D on Serum 25(OH)D Concentrations and Glycemic Control Indicators

Serum 25(OH)D concentrations were significantly increased in the EX + VD and VD groups, but they were not altered in the EX and Con groups (Figure 1b). No changes in weekly physical activity and sun exposure score during the interventions were observed. Compared with baseline, participants in the EX + VD group tended to have lower HOMA-IR scores (mean ± SD, 3.9 ± 3.0 vs. 3.0 ± 2.2; change%, −23.1%; effect size, 0.40; *p* = 0.086) and HbA1c levels (mean ± SD, 7.0 ± 1.9 vs. 6.3 ± 0.7%, change%, −10.0%; effect size, 0.46; *p* = 0.064) at endpoint, and the downregulating effect of EX + VD intervention on HbA1c levels tended to remain at the 12-week follow-up (Table 1, Tables S3 and S4). EX + VD and VD interventions significantly reduced fasting glucose levels by 11.9% and 14.4% (EX + VD, mean ±SD, 6.7 ± 1.4 vs. 5.9 ± 1.0; effect size, 0.65; VD, mean ±SD, 7.4 ± 1.9 vs. 6.5 ± 0.8; effect size,0.59; all *p*_s_ < 0.05) respectively, while EX intervention increased insulin secretion (5718 ± 717 vs. 6923 ± 1047; change %, 21.1%; effect size, 2.49; *p* = 0.027) (Table 1). There was no significant exercise and vitamin D interaction or any main effects on blood profiles (Supplementary Table S3). 

During the OGTT, compared with basal values, the levels of fasting glucose were relatively lower at five time points after EX + VD, VD, and EX interventions for 12 weeks. Opposite effects were seen for insulin concentrations. Such changes were not observed in the Con group (Supplementary Figure S1). 

### 3.3. Effects of Exercise and Vitamin D on Plasma Lipidome

**Figure 2:**
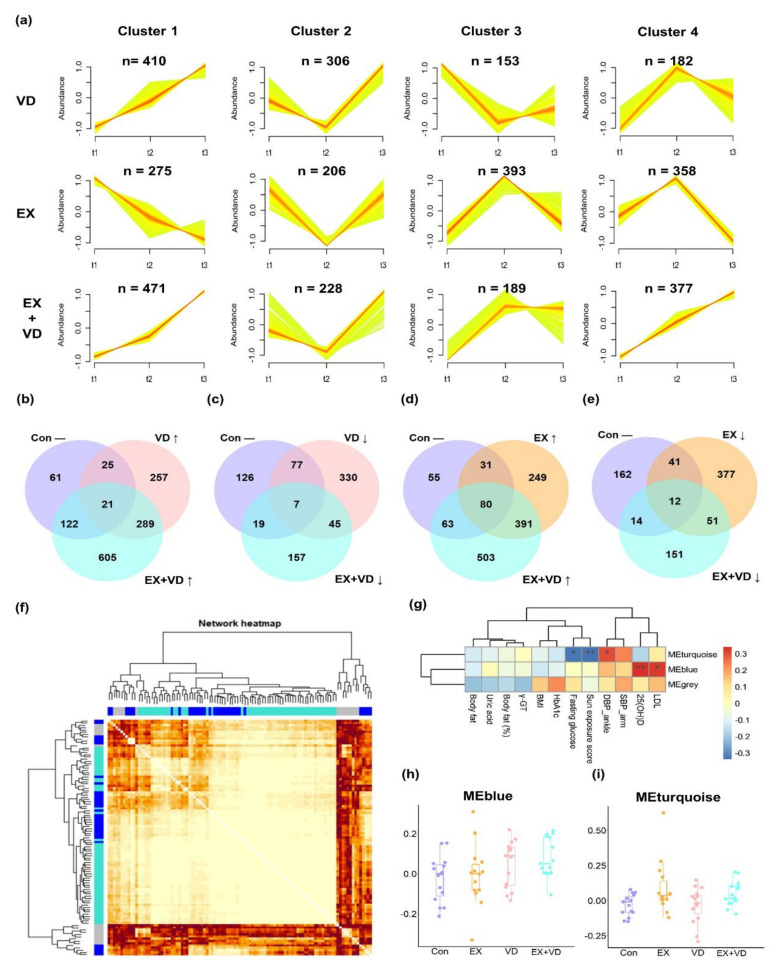
Plasma lipidome responding to interventions at three sampling time points. (**a**) The distinctive patterns showing different time-dependent changes in lipids of three intervention groups that were derived using the fuzzy c-means clustering algorithm with the time-serial data method. Numbers of lipid metabolites belonging to each cluster are displayed. (**b**–**e**) Venn diagrams showing large differences between the changed lipids in the VD, EX, and EX + VD groups compared with the Con group. (**f**) The co-expression network of plasma lipids generated by weighted correlation network analysis (WGCNA). The lipid species were hierarchically clustered using a dynamic tree-cutting algorithm with a minimum module size of ten. The resulting lipid modules were assigned color names (i.e., MEturquoise, MEblue, and MEgrey) and identified using the eigenvector of each module, designated the module eigenlipid (ME). The heatmap depicts the topological overlap matrix (TOM) among all lipids in the analysis. Each row and column in the clustering heat map corresponds to a lipid. A light color represents a low overlap with lipid expression pattern and a progressively darker red color represents a higher one. (**g**) Pearson’s correlations between the WGCNA derived modules and clinical indices). *, *p* < 0.05; **, *p* < 0.01. (**h**,**i**) The module scores of MEblue and MEturquoise differed between the VD, EX, and EX + VD groups with the Con group. EX + VD, exercise training and vitamin D supplementation group; VD, vitamin D supplementation group; EX, exercise training group. t1 and t2: EX + VD, *n* = 15; VD, *n* = 15; EX, *n* = 14; t3: EX + VD, *n* = 13; VD, *n* = 11; EX, *n* = 12.

A total of 1774 lipid features were identified and quantified, among which 1315 were annotated, including TGs (*n* = 280), PCs (*n* = 266), Cers (*n* = 134), sphingomyelins (SMs, *n* = 103), phosphatidylethanolamines (PEs, *n* = 80), diacylglycerols (DGs, *n* = 53), and lyso-phosphatidylcholines (LPCs, *n* = 38). To capture the overall perturbations in lipid features, 4 distinct clusters were determined for the VD, EX, and EX + VD groups using the fuzzy clustering algorithm, which represented how the relative abundances of lipids underwent changes with the different interventions (Figure 2a). The plasma levels of 410 lipid features in Cluster 1 were upregulated by VD intervention compared with baseline, and they were monotonically increased at the 12-week follow-up. In Cluster 2, levels of 306 lipid features were downregulated after VD intervention, which were then increased during the follow-up. A majority of lipid features were increased by EX intervention (Clusters 3 and 4, *n* = 393 and 358, respectively) and decreased subsequently at the 12-week follow-up. Conversely, EX intervention downregulated levels of 206 lipid features that were in turn increased at the 12-week follow-up. Of note, we found that levels of a total of 848 lipid features (Clusters 1 and 4) were upregulated by EX + VD intervention, which continued to increase until the 12-week follow-up. By contrast, EX + VD intervention seemed to reduce the levels of 228 lipid features, but they did not remain at the 12-week follow-up.

Among the lipid features, levels of 21 were increased by both VD and EX + VD interventions, while those of 7 were decreased (Figure 2b,c). Both EX and EX + VD interventions enhanced plasma levels of 80 lipid features while reducing those of 12 lipid features (Figure 2d,e). When assessed individually, levels of 32, 10, and 42 annotated lipids were notably altered by EX, VD, and EX + VD interventions, respectively (Supplementary Table S5). These mainly included 15 PCs, 12 LPCs, 7 dihexosyl N-acetylhexosyl ceramides (CerG2GNAc1s), 7 ganglioside monosialo trihexosyl ceramides (GM3s), 5 SMs, 4 PEs, 4 TGs, and 3 lyso-phosphatidylethanolamines (LPEs).

**Table 1:** Mean ± standard deviation (SD) of blood parameters from baseline (0-week) for each group.

Variables	EX + VD *n* = 15				VD *n* = 15				EX *n* = 14				Con *n* = 15				*p* _baseline_
	t1	t2	Δ%	*p*	t1	t2	Δ%	*p*	t1	t2	Δ%	*p*	t1	t2	Δ%	*p*	
Weight	68.9 ± 13.1	68.6 ± 13.8	−0.4%	0.560	71.4 ± 13.1	71.8 ± 12.6	0.6%	0.599	74.7 ± 15.1	73.6 ± 14.6	−1.5%	0.019	76.9 ± 10.9	76.7 ± 11.2	−0.3%	0.684	0.364
BMI (kg/m^2^)	25.1 ± 3.0	25.8 ± 4.1	2.8%	0.365	25.3 ± 3.3	25.4 ± 3.0	0.4%	0.668	26.1 ± 4.8	25.8 ± 4.6	−1.1%	0.036	27.2 ± 3.2	27.2 ± 3.3	0.0%	0.745	0.363
Body fat (%) ^a^	32.0 ± 6.1	31.0 ± 5.7	−3.1%	0.093	27.9 ± 9.2	28.4 ± 9.9	1.8%	0.438	30.9 ± 8.6	29.8 ± 8.7	−3.6%	0.020	30.3 ± 6.6	29.8 ± 6.0	−1.7%	0.174	0.532
Physical activity (MET-min/Week)	3164 ± 2413	4971 ± 4070	57.1%	0.138	4065 ± 4583	4225 ± 2722	3.9%	0.896	4334 ± 4286	4092 ± 5709	−5.6%	0.831	2801 ± 1976	3311 ± 1959	18.2%	0.221	0.596
Sun exposure weekly score	16.5 ± 8.3	16.4 ± 11.9	−0.6%	0.966	13.6 ± 4.1	19.3 ± 9.5	41.9%	0.082	16.1 ± 8.8	20.7 ± 11.4	28.6%	0.153	13.2 ± 7.3	18.8 ± 16.1	42.4%	0.212	0.506
Apolipoprotein A (g/L)	1.29 ± 0.30	1.31 ± 0.26	1.6%	0.711	1.29 ± 0.23	1.31 ± 0.26	1.6%	0.549	1.29 ± 0.22	1.30 ± 0.20	0.8%	0.802	1.18 ± 0.26	1.28 ± 0.21	8.5%	0.099	0.977
Apolipoprotein B (g/L)	0.83 ± 0.32	0.85 ± 0.26	2.4%	0.634	0.85 ± 0.19	0.83 ± 0.19	−2.4%	0.639	0.90 ± 0.25	0.93 ± 0.29	3.3%	0.468	0.73 ± 0.17	0.80 ± 0.15	9.6%	0.065	0.467
LDL-C (mmol/L)	2.3 ± 0.9	2.2 ± 0.7	−4.3%	0.587	2.3 ± 0.6	2.1 ± 0.6	−8.7%	0.105	2.5 ± 0.8	2.3 ± 0.7	−8.0%	0.059	2.1 ± 0.5	2.0 ± 0.5	−4.8%	0.192	0.590
HDL-C (mmol/L)	1.3 ± 0.4	1.3 ± 0.3	0.0%	0.242	1.2 ± 0.2	1.2 ± 0.3	0.0%	0.948	1.3 ± 0.3	1.3 ± 0.2	0.0%	0.830	1.2 ± 0.2	1.2 ± 0.2	0.0%	0.808	0.977
Cholesterol (mmol/L)	4.3 ± 1.2	4.4 ± 1.1	2.3%	0.787	4.3 ± 0.8	4.1 ± 0.8	−4.7%	0.327	4.7 ± 0.8	4.5 ± 0.8	−4.3%	0.207	4.0 ± 0.7	3.9 ± 0.6	−2.5%	0.498	0.326
Triglyceride (mmol/L)	1.4 ± 0.7	1.4 ± 0.8	0.0%	0.711	1.5 ± 0.7	1.5 ± 0.6	0.0%	0.990	2.0 ± 1.0	1.7 ± 0.7	−15.0%	0.239	1.3 ± 0.5	1.6 ± 0.7	23.1%	0.028	0.132
HbA1c (%)	7.0 ± 1.9	6.3 ± 0.7	−10.0%	0.064	6.9 ± 1.4	6.6 ± 0.8	−4.3%	0.318	6.7 ± 1.2	6.5 ± 1.1	−3.0%	0.316	7.2 ± 1.6	6.7 ± 0.8	−6.9%	0.186	0.835
Fasting glucose (mmol/L)	6.7 ± 1.4	5.9 ± 1.0	−11.9%	0.016	7.4 ± 1.9	6.5 ± 0.8	−12.2%	0.032	7.8 ± 2.1	7.2 ± 2.1	−7.7%	0.114	7.5 ± 2.6	7.1 ± 1.3	−5.3%	0.588	0.559
Fasting insulin (μU/mL)	12.6 ± 8.6	10.8 ± 6.2	−14.3%	0.389	9.7 ± 5.6	11.0 ± 8.9	13.4%	0.294	11.7 ± 6.1	12.1 ± 8.3	3.4%	0.809	14.7 ± 7.6	15.1 ± 11.0	2.7%	0.813	0.240
HOMA-IR	3.9 ± 3.0	3.0 ± 2.2	−23.1%	0.086	3.4 ± 2.6	3.2 ± 2.7	−5.9%	0.649	3.9 ± 2.3	3.8 ± 2.3	−2.6%	0.823	4.9 ± 2.6	5.1 ± 4.9	4.1%	0.814	0.384
Matsuda Index	2.9 ± 1.5	3.3 ± 1.9	13.8%	0.383	3.3 ± 2.1	3.3 ± 2.2	0.0%	0.993	2.8 ± 1.3	3.2 ± 2.1	14.3%	0.448	2.7 ± 2.0	2.5 ± 1.3	−7.4%	0.205	0.601
Glucose AUC ^b^	1621 ± 319	1542 ± 249	−4.9%	0.276	1681 ± 274	1541 ± 277	−8.3%	0.093	1728 ± 388	1653 ± 420	−4.3%	0.215	1672 ± 368	1642 ± 287	−1.8%	0.605	0.865
Insulin AUC ^b^	6353 ± 2277	6869 ± 2428	8.1%	0.506	5794 ± 1709	6812 ± 3050	17.6%	0.077	5718 ± 2683	6924 ± 3919	21.1%	0.027	7029 ± 2917	6930 ± 2530	−1.4%	0.862	0.458

Δ, change from the endpoint to baseline; EX + VD, exercise training and vitamin D supplementation group; VD, vitamin D supplementation group; EX, exercise training group; Con, control group; LDL-C, low-density lipoprotein cholesterol; HDL-C, high-density lipoprotein cholesterol; HbA1c, glycated hemoglobin; HOMA-IR, homeostasis model assessment of insulin resistance; AUC, area under the curve during oral glucose tolerance test. The increments in the AUC of glucose and insulin during the complete 120-min period of the OGTT were calculated using the trapezoid rule to assess glucose tolerance and insulin secretion. t1, time 1 (0-week); t2, time 2 (12-week). ^a^, *n* = 14 in Con; ^b^, *n* = 13 in VD were included.

We further clustered those lipid features into co-expression modules to identify those with similar physiological and molecular characteristics, and 90 out of 105 lipids were successfully clustered into 2 modules (Figure 2f,g and Supplementary Table S6). Compared with the Con group, the EX + VD group showed higher levels of the WGCNA-derived eigenlipid module (MEblue), which mainly included long-chain TGs with unsaturated fatty acyls (TG [56:8], TG [52:4], TG [56:7]), and the levels tended to be higher than those in the VD or EX group (Figure 2h). Both the EX and EX + VD groups had higher levels of the eigenlipid module (MEturquoise) dominated by LPCs than the Con group, suggesting that the regulating effects on LPCs could be possibly driven by EX intervention (Figure 2i). Of note, WGCNA-derived lipid patterns were significantly correlated with clinical indices that were particularly improved by the designed health-promoting interventions, i.e., 25(OH)D, LDL-C, DBP, and fasting glucose (Figure 2g). In addition, we found that modulated lipids in the EX + VD group were significantly correlated with obesity-related traits (Supplementary Figure S2). For instance, the EX + VD group showed increased levels of GM3 (36:2) (r = −0.34, *p* = 0.01), PCs (8:0/10:0) (r = −0.27, *p* = 0.04), and LPC (16:0) (r = −0.32, *p* = 0.02), which were negatively correlated with body fat.

**Figure 3:**
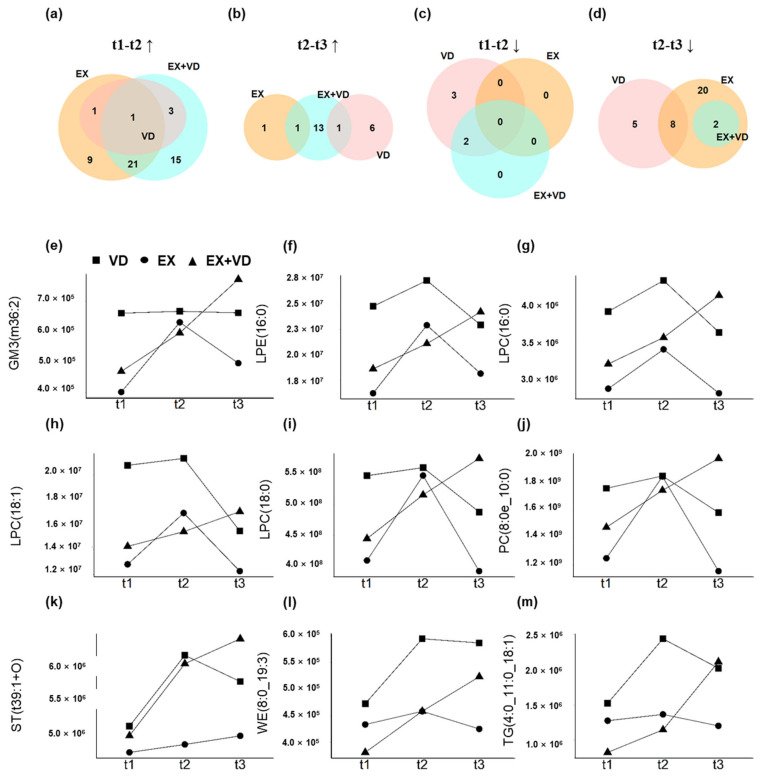
Alterations in plasma lipidome with interventions at three sampling time points. (**a**–**d**) Venn diagrams showing substantial differences in the changes of 77 lipids (t1 vs. t2) between VD, EX, and EX + VD groups. (**e**–**m**) Representative changed lipids with the synergistic effect of EX + VD intervention. EX + VD, vitamin D supplementation and exercise training group; VD, vitamin D supplementation group; EX, exercise training group. t1 and t2: EX + VD, *n* = 15; VD, *n* = 15; EX, *n* = 14; t3: EX + VD, *n* = 13; VD, *n* = 11; EX, *n* = 12.

Most interestingly, we observed the regulatory effects of EX + VD intervention on certain plasma lipids, which could last until the 12-week follow-up (Figure 3) and aligned with the results of fuzzy clustering (Figure 2a). Herein, we identified that levels of GM3 (36:2), LPE (16:0), LPC (16:0), LPC (18:1), LPC (18:0), PCs (8:0/10:0), sterol (ST, 39:1), wax esters (WEs, 8:0/19:3), and TGs (4:0/11:0/18:1) were progressively increased by EX + VD intervention until the 12-week follow-up. Such effects were not seen in the EX or VD group during intervention (Figure 3e–m).

### 3.4. Individual Responses to Interventions Improving Glycemic Control and Baseline Prediction 

**Figure 4:**
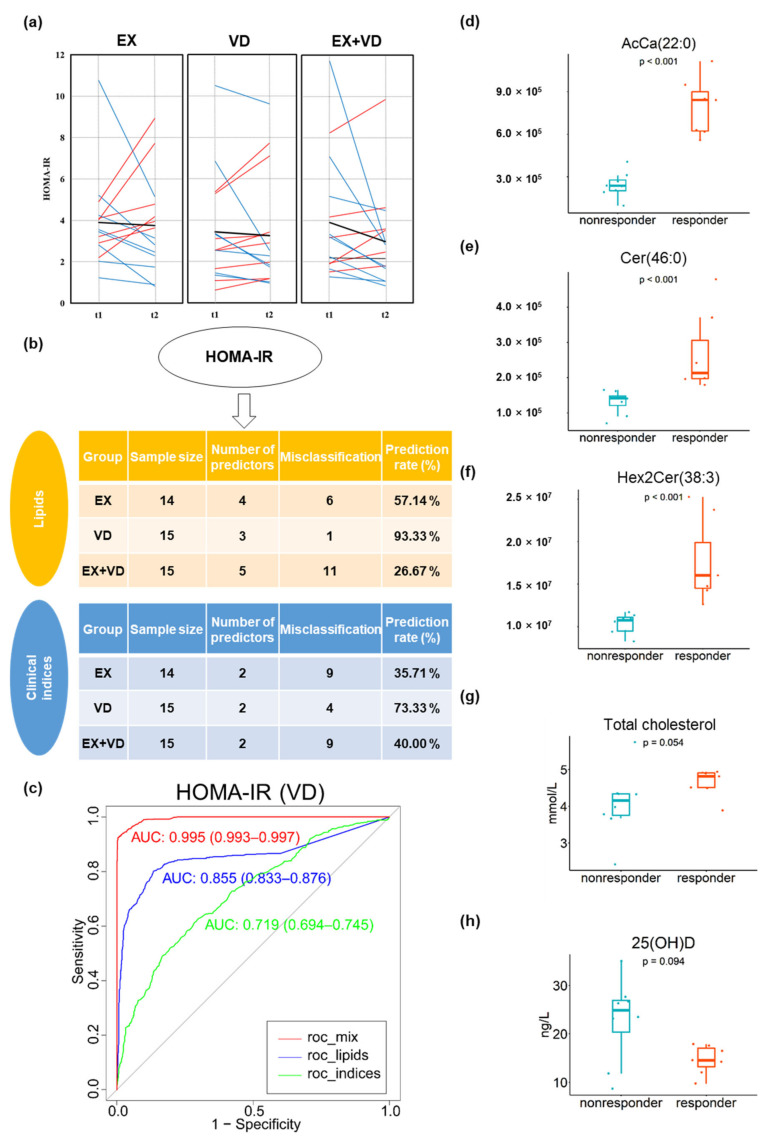
Responders and non-responders to interventions and baseline prediction for HOMA-IR. (**a**) Individual variations in HOMA-IR changes in EX, VD, and EX + VD groups. Blue lines indicate individuals with decreased values. Red lines indicate individuals with increased values. Black normal lines indicate individuals with no changed level of HOMA-IR. Black bold lines indicate the change in means. (**b**) Baseline prediction based on random forest algorithm incorporated into a repeated double cross-validation framework with unbiased variable selection in the EX, VD, and EX + VD groups, respectively. Optimal panels of markers that could predict HOMA-IR were identified. (**c**) Prediction accuracy was determined by misclassification rate % and area under receiver operating characteristic curve (AROC, 95% confidence interval). (**d**–**h**) Baseline levels of predictors of HOMA-IR in VD group differed between responders and non-responders. Wilcoxon test was conducted with *p* < 0.05 considered a significant difference. AcCa, acyl carnitine; Cer, ceramides; HexCer1/Hex2Cer, hexosyl ceramide; EX + VD, exercise training and vitamin D supplementation group; VD, vitamin D supplementation group; EX, exercise training group. t1 and t2: EX + VD*, n* = 15; VD, *n* = 15; EX, *n* = 14; t3: EX + VD, *n* = 13; VD, *n* = 11; EX, *n* = 12.

There was high inter-individual variability in multiple glycemic parameters in response to the interventions, which might have contributed to the lack of benefit of the VD and EX interventions and their combination on glycemic control, i.e., HOMA-IR in participants with T2DM (Figure 4a, Supplementary Figure S3a–c ). This greatly raised our interest in assessing the responsiveness to each parameter and evaluating whether clinical and anthropometrical parameters as well as plasma lipids at baseline could be used to predict the effects of intervention. There were 7, 8, 9, and 4 participants who benefitted from VD intervention with regard to HOMA-IR, HbA1c level, glucose tolerance, and insulin secretion, respectively, all of whom were considered as responders. In the EX group, there were 8, 7, 10, and 5 responders, and there were 8, 9, 10, and 7 responders in the EX + VD group, respectively.

We conducted comprehensive machine learning analyses to identify baseline lipids that were associated with responsiveness and verified the model performance (Figure 4 and Supplementary Figure S3). Basal lipids were found to be associated with intervention effects and generally provided additional prediction power beyond the use of anthropometric and biomedical parameters. Specifically, baseline levels of three lipids, i.e., Cer (46:0), AcCa (22:0), and Hex2Cer (38:3), greatly predicted responders for HOMA-IR in the VD group (prediction rate = 93.3%, AROC = 0.855, 95% CI [0.833, 0.876], Figure 4b,c), which significantly outperformed clinical indices (prediction rate = 73.3%, AROC = 0.719, 95% CI [0.694, 0.745]). This finding suggested that participants who had higher baseline levels of these three lipids may benefit from VD supplementation and had reduced HOMA-IR scores after VD intervention compared with baseline. Moreover, the best prediction performance was consistently achieved in the presence of these lipid predictors for the responsiveness of HbA1c levels (AROC = 0.802, 95% CI [0.779, 0.825]) in the EX group, and the prediction performance was largely improved when these lipid predictors were added to clinical indices (i.e., HbA1c, AROC = 0.993 [0.991–0.996]; glucose AUC, AROC = 0.920 [0.906–0.934]), compared to using either lipid or clinical indices alone (Supplementary Figures S3 and S4). 

## 4. Discussion

In this randomized placebo-controlled trial, the overall beneficial effects of a 12-week endurance exercise training program with and without VD intervention on fasting glucose in participants with T2DM were observed, accompanied by notable alterations in the plasma lipidome. Although VD intervention failed to provide additional benefits to the HOMA-IR index and other glycemic indicators beyond EX intervention, we observed a regulatory effect of EX + VD intervention on the plasma lipidome and noted that such effects lasted until the 12-week follow-up. Some specific lipid changes were associated with changes in obesity-related traits. Importantly, we found high inter-individual variability in glycemic control indicators, i.e., HOMA-IR, HbA1c level, and glucose tolerance, as a result of our interventions, which to a great extent may explain the lack of synergistic improvement in glycemic control upon intervention. We thereafter identified several lipid species alone or in combination with clinical indices that largely improved the prediction of changes in glycemic control, with an AROC value over 0.9.

Recently, a systematic review reported that combined exercise training (endurance and resistance exercises) was the most effective exercise intervention for cardiometabolic outcomes in participants with overweight and obesity [31]. However, regarding weight loss, another review stated that there was no difference among combined, endurance, and resistance exercises in participants with T2DM [32]. Moreover, accumulating evidence reported that endurance exercise was more likely to be associated with greater BMI reduction and had a greater effect on the improvement of glucose control than resistance exercise [3,4,5,6]. In the current study, the mean BMI of included participants was 25.9 kg/m^2^ with an SD of 3.6 at baseline, and they were identified as overweight and obese; thus, we chose endurance exercise as it is more popular in China [33]. 

Hyperglycemia is a key risk factor for the development of diabetic complications [34]. Vitamin D can directly activate the AMPK-GLUT-4 signaling through the vitamin D receptor, thereby promoting glucose utilization during exercise [9]. However, limited human studies have been conducted to investigate effects of exercise training complemented with vitamin D supplementation on glycemic control, and the results were inconsistent [35,36]. In a 12-week intervention conducted with 48 middle-aged men with T2DM, adding vitamin D greatly outperformed resistance training alone in improving glucose homeostasis [36]. In the present study, we noticed a within-group beneficial improvement in fasting glucose and insulin secretion for T2DM participants who underwent EX + VD or EX intervention, while the effects on glycemic control indicators were inconsistent. VD intervention failed to provide added value beyond EX intervention alone to glycemic control indicators, including HOMA-IR. Our findings are in agreement with a study conducted with 42 elderly women with T2DM in which the effects of 12-week circuit training and vitamin D intervention on fasting glucose levels and HOMA-IR index were assessed [35]. The concurrent impacts of exercise and vitamin D supplementation on hyperglycemia improvement warrant further study in large-scale RCTs. 

Recent advances in lipidomics have greatly expanded the associations between lipid species and metabolic pathways with disease onset and progression, such as T2DM [15,16]. Elevated fasting blood triglyceride levels have been considered an independent risk factor for T2DM. However, recent studies have revealed that triglycerides containing different proportions of saturated, monounsaturated, or polyunsaturated fatty acyls could exhibit different biological functions involved in the pathophysiology of T2DM [37,38,39]. In a primary preventive cohort including 685 individuals, TGs consisting of short-chain saturated fatty acyl chains were found to be associated with higher risk of cardiovascular disease development [40]. A lacto-ovo-vegetarian diet has been shown to increase plasma long-chain unsaturated TG levels while decreasing those of saturated fatty acyls, resulting in a lowered atherosclerotic burden in participants with coronary artery disease [41]. 

Moreover, maintaining appropriate levels of exercise as an effective strategy for keeping healthy can produce vast changes in multiple lipid species [42]. Endurance exercise has previously been shown to substantially increase levels of plasma TGs containing long-chain fatty acids, e.g., TG(47:1), TG(51:1), and TG(55:3) [43]. Consistently, our study supported the upregulating effects of exercise with and without vitamin D supplementation on levels of long-chain unsaturated TGs, which were positively related to serum 25(OH)D concentrations (increased by at least 36.3% after vitamin D supplementation). In addition, we found that exercise training significantly increased levels of highly unsaturated glycolipids such as DGDG, and the effect remained at the 12-week follow-up. DGDG has previously been shown to participate in inhibiting fat accumulation and has anti-inflammatory activity [44,45]. 

Glycerophospholipids, in particular PCs and LPCs, play important roles in regulating glucose homeostasis and chronic inflammatory status [38,39,46,47,48,49], but the mechanisms remain largely unexplored [39]. In our study, EX intervention with and without VD intervention increased levels of LPCs such as C20:3, C19:0, C20:0, C18:1, and C20:4, as well as PCs such as C20:2, C18:0, C40:7, C32:1, and C19:2, and such effects seemed to last during follow-up. Previous studies have identified certain LPCs and PCs as biomarkers capable of discriminating T2DM participants from healthy controls. A recent study reported that aerobic and strength exercises for 8 weeks significantly upregulated levels of plasma LPC (16:0), LPC (18:0), and LPC (20:2) in women with obesity [50]. LPC (14:0/18:0/18:1) treatment was found to stimulate glucose uptake via PI3-kinase-independent protein kinase C δ (PKCδ) activation in a mouse diabetic model in a dose-dependent manner [51]. We also found increased levels of CerG2GNAc1s (i.e., 38:4, 32:1, 36:1) induced by EX or EX + VD intervention. CerG2GNAc1s belonging to sphingolipids exhibit various biological functions [52]. Sphingolipids such as ceramides, glycosphingolipids, and sphingomyelin are important molecules that regulate critical cellular functions and are considered putative nutritional intermediates linking obesity to diabetes risk [53,54]. Despite considerable attention being devoted to the question of how ceramides and its derivatives are mechanically involved in developing metabolic disease, there exist enormous gaps in knowledge that warrant further investigations.

Apart from the observed effects of EX and VD interventions on glycemic control and the plasma lipidome, we found that the individual responsiveness of glycemic control indicator changes varied greatly among those undergoing interventions. For participants allocated in each intervention group, only about half were responders for HOMA-IR, HbA1c level, and glucose tolerance (i.e., participants who had lower glycemic indicators after intervention compared with baseline). These results corroborate and expand existing evidence that there are inter-individual differences in responses to diet or exercise treatments [18,19], highlighting the necessity and importance of personalized exercise programs or in combination with nutrition intervention [20,21]. Most importantly, the responsiveness of intervention-derived improvements in glycemic parameters at baseline could be predicted with high accuracy, with an AROC value over 0.90, by applying machine learning-selected lipid and clinical predictors. In addition to higher body fat and total cholesterol, participants with higher levels of Cer (46:0), PC (37:4), and GM3 (33:5) were more likely to have reduced glycemic values (HOMA-IR and HbA1c) than their counterparts. 

Our findings explore the use of metabolomics in the fields of exercise metabolism and sports nutrition, promoting personalized exercise and/or nutrition strategies to maximize exercise performance and provide additional exercise-related health benefits, such as glycemic control. Additionally, no intervention-related adverse events were reported. These results indicated progressive and individual tailored programming may be effective in controlling body weight in participants with T2DM in a gym setting. Notably, although the frequency of attendance at exercise sessions did not differ between the EX and EX + VD groups, lower levels of adherence of some participants to the exercise program in the present study may have underestimated the effect of exercise on glycemic indicators, resulting in the inability to achieve significant results for blood indicators. Further comprehensive interventions targeting lifestyle, using novel approaches such as mHealth, are warranted to address this issues.

Our study had several limitations. First, the study duration was relatively short, although it has been demonstrated that body fat and blood metabolic profile changes can be detected within 12 weeks [55]. Second, the relatively small group sizes in our study may have limited our power to detect profound differential lipid species changes, although the pre-power analysis showed that the sample size in our study would provide a more than 85% chance to demonstrate the effect on HOMA-IR. Third, we used age-predicted HRmax instead of HRmax measured during maximal exercise tests. Despite incorporating the RPE to assist tracking exercise intensity with age-predicted HR, there still remained a potential for bias. Furthermore, maximal or submaximal testing was encouraged to evaluate intervention efficacy and exercise intensity. Finally, this study did not comprehensively assess dietary intake, although participants were instructed not to alter their diet; these factors could have affected the results. Thus, future studies should attempt to thoroughly control the diet. However, a strength of our study is that given the rising prevalence and poor control of T2DM worldwide, we believe that this is the first RCT designed with exercise and/or vitamin D supplementation as a two-factorial intervention for 12 weeks, followed by another evaluation at 12 weeks to determine the underlying mechanism. Our findings provide some novel evidence for related metabolic disease management from the perspective of lifestyle adjustments.

## 5. Conclusions

Although vitamin D supplementation failed to provide an additive effect beyond exercise on glucose homeostasis, we observed a regulatory effect of exercise combined with vitamin D intervention on the plasma lipidome. Such effects lasted until the 12-week follow-up in the EX + VD group, but this was not the case for the EX and VD groups. Moreover, the consideration of several lipid species at baseline could largely improve the prediction of changes in glycemic control rather than traditional clinical indices. Collectively, our data provide practical evidence that can be applied to exercise- and nutrient-specific conditioning programs for T2DM prevention and control. 

## Data Availability

The data used in this study are not publicly available due to ethical reasons; the corresponding author can provide further information on these data upon reasonable request.

## Supplementary Materials

The following supporting information can be downloaded at: https://www.mdpi.com/article/10.3390/nu15133027/s1. Supplementary Figure S1. Blood glucose and insulin during oral glucose tolerance test according to treatment group before and after intervention in 55 T2DM participants #. The values are expressed as mean ± standard deviation of blood glucose and insulin at 30, 60, 90, and 120 min during OGTT. #, EX + VD, *n* = 15; VD, *n* = 13; EX, *n* = 14; Con, *n* = 13. *, *p* < 0.05 compared with values at the same time points using paired *t*-test. Supplementary Figure S2. The correlations between metabolite changes and clinical index changes from baseline (0-week) among 59 participants. The clinical parameters were selected based on their associations with vitamin D metabolism (liver and kidney function indicators, sun exposure, physical activity), storage tissues (fat- or muscle-related indicators), and glucose-related indicators (blood pressure and lipids).*, *p* < 0.05; **, *p* < 0.01; ***, *p* < 0.001; EX + VD, *n* = 15; VD, *n* = 15; EX, *n* = 14; Con, *n* = 15. Supplementary Figure S3. Responders and non-responders to interventions and baseline prediction for glycemic control indicators. (a–c) Individual variations in HbA1c, glucose AUC, and insulin AUC changes in EX and EX + VD groups. Blue lines indicate individuals with decreased values. Red lines indicate individuals with increased values. Black normal lines indicate individuals with no changed level of the corresponding parameter. Black bold lines indicate the change in means. (d) Baseline prediction based on random forest algorithm incorporated into a repeated double cross-validation framework with unbiased variable selection in VD, EX, and EX + VD groups, respectively. Optimal panel of markers that could predict HbA1c, glucose AUC, and insulin AUC was identified. Prediction accuracy was determined by misclassification rate % and area under receiver operating characteristic curve (AROC, 95% confidence interval). EX + VD, vitamin D supplementation and exercise training group; VD, vitamin D supplementation group; EX, exercise training group. t1 and t2: EX + VD, *n* = 15; VD, *n* = 15; EX, *n* = 14; t3: EX + VD, *n* = 13; VD, *n* = 11; EX, *n* = 12. Supplementary Figure S4. Baseline levels of predictors differed between responders and non-responders. (a,b) Baseline levels of predictors of HbA1c in EX group differed between responders and non-responders. (c–f) Baseline levels of predictors of glucose AUC in EX group differed between responders and non-responders. (g–j) Baseline levels of predictors of insulin AUC in VD group differed between responders and non-responders. (k,l) Baseline levels of clinical indices of glucose AUC in EX group differed between responders and non-responders. Wilcoxon test was conducted with *p* < 0.05 considered a significant difference. PC, phosphatidylcholine; GM3, ganglioside monosialo trihexosyl ceramide; TGs, triglycerols; Cer, ceramides; HexCer1/Hex2Cer, hexosyl ceramide; MGDG, monogalactosyldiacylglycerol; EX + VD, exercise training and vitamin D supplementation group; VD, vitamin D supplementation group; EX, exercise training group. EX + VD, *n* = 15; VD, *n* = 15; EX, *n* = 14. Supplementary Table S1. The protocol of endurance exercise training program. Supplementary Table S2. Procedures of lipidomic measurement. Supplementary Table S3. Mean differences (95% CI) of blood parameters from baseline (0-week) with each group using 2 × 2 factorial ANOVA. Supplementary Table S4. Mean differences of glycemic control indicators at three time points for each group. Supplementary Table S5. The 77 lipids that had pronounced changes between t2 and t1 sampling in the VD, EX, and EX + VD groups and their corresponding module colors of WGCNA. Supplementary Table S6. Different co-expression modules of lipids from WGCNA.

## References

[1] Mortality G.B.D. (2015). Causes of Death, C. Global, Regional, and National Age-Sex Specific all-Cause and Cause-Specific Mortality for 240 Causes of Death, 1990–2013: A Systematic analysis for the Global Burden of Disease Study 2013. Lancet.

[2] Wang L., Peng W., Zhao Z., Zhang M., Shi Z., Song Z., Zhang X., Li C., Huang Z., Sun X. (2021). Prevalence and Treatment of Diabetes in China, 2013–2018. JAMA.

[3] Kanaley J.A., Colberg S.R., Corcoran M.H., Malin S.K., Rodriguez N.R., Crespo C.J., Kirwan J.P., Zierath J.R. (2022). Exercise/Physical Activity in Individuals with Type 2 Diabetes: A Consensus Statement from the American College of Sports Medicine. Med. Sci. Sport. Exerc..

[4] Colberg S.R., Sigal R.J., Yardley J.E., Riddell M.C., Dunstan D.W., Dempsey P.C., Horton E.S., Castorino K., Tate D.F. (2016). Physical Activity/Exercise and Diabetes: A Position Statement of the American Diabetes Association. Diabetes Care.

[5] National Fitness Guide. General Administration of Sport of China. https://www.sport.gov.cn/n315/n331/n405/c819327/content.html.

[6] Chinese Diabetes Society Chinese Guidelines of Exercise Therapy in Diabetes Mellitus. https://diab.cma.org.cn/cn/zhinangongshi.aspx.

[7] Janssen S.M., Connelly D.M. (2021). The effects of exercise interventions on physical function tests and glycemic control in adults with type 2 diabetes: A systematic review. J. Bodyw. Mov. Ther..

[8] Sampath K.A., Maiya A.G., Shastry B.A., Vaishali K., Ravishankar N., Hazari A., Gundmi S., Jadhav R. (2019). Exercise and insulin resistance in type 2 diabetes mellitus: A systematic review and meta-analysis. Ann. Phys. Rehabil. Med..

[9] Manna P., Jain S.K. (2012). Vitamin D up-regulates glucose transporter 4 (GLUT4) translocation and glucose utilization mediated by cystathionine-gamma-lyase (CSE) activation and H2S formation in 3T3L1 adipocytes. J. Biol. Chem..

[10] Antoniak A.E., Greig C.A. (2017). The effect of combined resistance exercise training and vitamin D3 supplementation on musculoskeletal health and function in older adults: A systematic review and meta-analysis. BMJ Open.

[11] Sun X., Cao Z.B., Tanisawa K., Ito T., Oshima S., Higuchi M. (2014). The Relationship between Serum 25-Hydroxyvitamin D Concentration, Cardiorespiratory Fitness, and Insulin Resistance in Japanese Men. Nutrients.

[12] Goldberg I.J. (2001). Clinical review 124—Diabetic dyslipidemia: Causes and consequences. J. Clin. Endocr. Metab..

[13] Andersen C.J. (2022). Lipid Metabolism in Inflammation and Immune Function. Nutrients.

[14] Taskinen M.R., Boren J. (2015). New insights into the Pathophysiology of Dyslipidemia in Type 2 Diabetes. Atherosclerosis.

[15] Meikle P.J., Wong G., Barlow C.K., Weir J.M., Greeve M.A., MacIntosh G.L., Almasy L., Comuzzie A.G., Mahaney M.C., Kowalczyk A. (2013). Plasma Lipid Profiling Shows Similar Associations with Prediabetes and Type 2 Diabetes. PLoS ONE.

[16] Miao G., Zhang Y., Huo Z., Zeng W., Zhu J., Umans J.G., Wohlgemuth G., Pedrosa D., DeFelice B., Cole S.A. (2021). Longitudinal Plasma Lipidome and Risk of Type 2 Diabetes in a Large Sample of American Indians with Normal Fasting Glucose: The Strong Heart Family Study. Diabetes Care.

[17] Monnerie S., Comte B., Ziegler D., Morais J.A., Pujos-Guillot E., Gaudreau P. (2020). Metabolomic and Lipidomic Signatures of Metabolic Syndrome and its Physiological Components in Adults: A Systematic Review. Sci Rep..

[18] Brennan A.M., Standley R.A., Yi F., Carnero E.A., Sparks L.M., Goodpaster B.H. (2020). Individual Response Variation in the Effects of Weight Loss and Exercise on Insulin Sensitivity and Cardiometabolic Risk in Older Adults. Front. Endocrinol..

[19] Al-Daghri N.M., Mohammed A.K., Al-Attas O.S., Ansari M.G.A., Wani K., Hussain S.D., Sabico S., Tripathi G., Alokail M.S. (2017). Vitamin D Receptor Gene Polymorphisms Modify Cardiometabolic Response to Vitamin D Supplementation in T2DM Participants. Sci. Rep..

[20] Garcia-Perez I., Posma J.M., Chambers E.S., Mathers J.C., Draper J., Beckmann M., Nicholson J.K., Holmes E., Frost G. (2020). Dietary Metabotype Modelling Predicts Individual Responses to Dietary Interventions. Nat. Food.

[21] Palmnas M., Brunius C., Shi L., Rostgaard-Hansen A., Torres N.E., Gonzalez-Dominguez R., Zamora-Ros R., Ye Y.L.Q., Halkjaer J., Tjonneland A. (2020). Perspective: Metabotyping-A Potential Personalized Nutrition Strategy for Precision Prevention of Cardiometabolic Disease. Adv. Nutr..

[22] Sun X., Cao Z.B., Tanisawa K., Ito T., Oshima S., Higuchi M. (2016). Vitamin D Supplementation Reduces Insulin Resistance in Japanese Adults: A Secondary Analysis of a Double-Blind, Randomized, Placebo-Controlled Trial. Nutr. Res..

[23] Faul F., Erdfelder E., Lang A.G., Buchner A. (2007). G*Power 3: A Flexible Statistical Power Analysis Program for the Social, Behavioral, and Biomedical Sciences. Behav. Res. Methods.

[24] Tanaka H., Monahan K.D., Seals D.R. (2001). Age-Predicted Maximal Heart Rate Revisited. J. Am. Coll. Cardiol..

[25] Sun X., Xiao W., Li Z., Zhou S., Dong M., Huang C., Ma Y., Gou B. (2022). Does Vitamin D Supplementation Improve Bone Health, Body Composition and Physical Performance Beyond Endurance Exercise in Participants with Type 2 Diabetes: A secondary Analysis of randomized controlled trial. Front. Physiol..

[26] Craig C.L., Marshall A.L., Sjostrom M., Bauman A.E., Booth M.L., Ainsworth B.E., Pratt M., Ekelund U., Yngve A., Sallis J.F. (2003). International Physical Activity Questionnaire: 12-Country Reliability and Validity. Med. Sci. Sports Exerc..

[27] Hanwell H.E., Vieth R., Cole D.E., Scillitani A., Modoni S., Frusciante V., Ritrovato G., Chiodini I., Minisola S., Carnevale V. (2010). Sun Exposure Questionnaire Predicts Circulating 25-Hydroxyvitamin D concentrations in Caucasian Hospital Workers in Southern Italy. J. Steroid. Biochem. Mol. Biol..

[28] Matthews D.R., Hosker J.P., Rudenski A.S., Naylor B.A., Treacher D.F., Turner R.C. (1985). Homeostasis Model Assessment—Insulin Resistance and Beta-Cell Function from Fasting Plasma-Glucose and Insulin Concentrations in Man. Diabetologia.

[29] Matsuda M., DeFronzo R.A. (1999). Insulin Sensitivity Indices Obtained from Oral Glucose TOLERANCE testing: Comparison with the Euglycemic Insulin Clamp. Diabetes Care.

[30] Wolever T.M.S. (2004). Effect of Blood Sampling Schedule and Method of Calculating the Area under the Curve on Validity and Precision of Glycaemic Index Values. Br. J. Nutr..

[31] Batrakoulis A., Jamurtas A.Z., Metsios G.S., Perivoliotis K., Liguori G., Feito Y., Riebe D., Thompson W.R., Angelopoulos T.J., Krustrup P. (2022). Comparative Efficacy of 5 Exercise Types on Cardiometabolic Health in Overweight and Obese Adults: A Systematic Review and Network Meta-Analysis of 81 Randomized Controlled Trials. Circ. Cardiovasc. Qual. Outcomes.

[32] Pan B., Ge L., Xun Y.Q., Chen Y.J., Gao C.Y., Han X., Zuo L.Q., Shan H.Q., Yang K.H., Ding G.W. (2018). Exercise Training Modalities in Patients with type 2 Diabetes Mellitus: A Systematic Review and Network Meta-Analysis. Int. J. Behav. Nutr. Phys. Act..

[33] Li Y.-M., Han J., Liu Y., Wang R., Wang R., Wu X.P., Cao Z.B. (2019). China Survey of Fitness Trends for 2020. ACSM’s Health Fit. J..

[34] Woerle H.J., Neumann C., Zschau S., Tenner S., Irsigler A., Schirra J., Gerich J.E., Goke B. (2007). Impact of Fasting and Postprandial glycemia on Overall Glycemic Control in Type 2 Diabetes Importance of Postprandial Glycemia to Achieve Target HbA1c Levels. Diabetes Res. Clin. Pract..

[35] Kim H.J., Kang C.K., Park H., Lee M.G. (2014). Effects of Vitamin D Supplementation and circuit Training on Indices of Obesity and Insulin Resistance in T2D and Vitamin D Deficient Elderly Women. J. Exerc. Nutr. Biochem..

[36] Dadrass A., Mohamadzadeh Salamat K., Hamidi K., Azizbeigi K. (2019). Anti-Inflammatory Effects of Vitamin D and Resistance Training in Men with Type 2 Diabetes Mellitus and Vitamin D Deficiency: A Randomized, Double-Blinded, Placebo-Controlled Clinical Trial. J. Diabetes Metab. Disord..

[37] Liu P.P., Zhu W., Chen C., Yan B., Zhu L., Chen X., Peng C. (2020). The Mechanisms of Lysophosphatidylcholine in the Development of Diseases. Life Sci..

[38] Zhao X.J., Fritsche J., Wang J.S., Chen J., Rittig K., Schmitt-Kopplin P., Fritsche A., Haring H.U., Schleicher E.D., Xu G.W. (2010). Metabonomic Fingerprints of Fasting Plasma and Spot Urine Reveal Human pre-Diabetic Metabolic Traits. Metabolomics.

[39] Barber M.N., Risis S., Yang C., Meikle P.J., Staples M., Febbraio M.A., Bruce C.R. (2012). Plasma Lysophosphatidylcholine Levels Are Reduced in Obesity and Type 2 Diabetes. PLoS ONE.

[40] Stegemann C., Pechlaner R., Willeit P., Langley S.R., Mangino M., Mayr U., Menni C., Moayyeri A., Santer P., Rungger G. (2014). Lipidomics Profiling and Risk of Cardiovascular Disease in the Prospective Population-Based Bruneck Study. Circulation.

[41] Djekic D., Shi L., Calais F., Carlsson F., Landberg R., Hyotylainen T., Frobert O. (2020). Effects of a Lacto-Ovo-Vegetarian Diet on the Plasma Lipidome and Its Association with Atherosclerotic Burden in Participants with Coronary Artery Disease—A Randomized, Open-Label, Cross-over Study. Nutrients.

[42] Belhaj M.R., Lawler N.G., Hoffman N.J. (2021). Metabolomics and Lipidomics: Expanding the Molecular Landscape of Exercise Biology. Metabolites.

[43] Latino F., Cataldi S., Carvutto R., De Candia M., D’Elia F., Patti A., Bonavolonta V., Fischetti F. (2021). The Importance of Lipidomic Approach for Mapping and Exploring the Molecular Networks Underlying Physical Exercise: A Systematic Review. Int. J. Mol. Sci..

[44] Yang Y.H., Du L., Hosokawa M., Miyashita K. (2020). Effect of Spirulina Lipids on High-Fat and High-Sucrose Diet induced Obesity and Hepatic Lipid Accumulation in C57BL/6J Mice. J. Funct. Foods.

[45] Gao Q.M., Yu K., Xia Y., Shine M.B., Wang C., Navarre D., Kachroo A., Kachroo P. (2014). Mono-and Digalactosyldiacylglycerol lipids Function Nonredundantly to Regulate Systemic Acquired Resistance in Plants. Cell Rep..

[46] Xu Y. (2002). Sphingosylphosphorylcholine and lysophosphatidylcholine: G protein-Coupled Receptors and Receptor-Mediated Signal Transduction. BBA-Mol. Cell Biol. Lipids.

[47] Han M.S., Lim Y.M., Quan W., Kim J.R., Chung K.W., Kang M., Kim S., Park S.Y., Han J.S., Park S.Y. (2011). Lysophosphatidylcholine as an Effector of Fatty acid-Induced Insulin Resistance. J. Lipid Res..

[48] Meikle P.J., Summers S.A. (2017). Sphingolipids and Phospholipids in Insulin Resistance and Related Metabolic Disorders. Nat. Rev. Endocrinol..

[49] Al-Sulaiti H., Diboun I., Agha M.V., Mohamed F.F.S., Atkin S., Domling A.S., Elrayess M.A., Mazloum N.A. (2019). Metabolic Signature of Obesity-Associated Insulin Resistance and Type 2 Diabetes. J. Transl. Med..

[50] San Martin R., Brandao C.F.C., Junqueira-Franco M.V.M., Junqueira G.P., de Freitas E.C., de Carvalho F.G., Rodrigues C.H.P., Aguesse A., Billon-Crossouard S., Krempf M. (2022). Untargeted Lipidomic Analysis of Plasma from Obese Women Submitted to Combined Physical Exercise. Sci Rep..

[51] Yea K., Kim J., Yoon J.H., Kwon T., Kim J.H., Lee B.D., Lee H.J., Lee S.J., Kim J.I., Lee T.G. (2009). Lysophosphatidylcholine Activates Adipocyte Glucose Uptake and Lowers Blood Glucose Levels in Murine Models of Diabetes. J. Biol. Chem..

[52] Russo D., Capolupo L., Loomba J.S., Sticco L., D’Angelo G. (2018). Glycosphingolipid Metabolism in Cell Fate Specification. J. Cell Sci..

[53] Shayman J.A. (2018). Targeting Glucosylceramide Synthesis in the Treatment of Rare and Common Renal Disease. Semin. Nephrol..

[54] Chavez J.A., Siddique M.M., Wang S.T., Ching J.H., Shayman J.A., Summers S.A. (2014). Ceramides and Glucosylceramides Are Independent Antagonists of Insulin Signaling. J. Biol. Chem..

[55] Cao L., Jiang Y., Li Q., Wang J., Tan S. (2019). Exercise Training at Maximal Fat Oxidation Intensity for Overweight or Obese Older Women: A Randomized Study. J. Sports Sci. Med..

