# Supplementary material for: Effects of Endurance Exercise and Vitamin D Supplementation on Insulin Resistance and Plasma Lipidome in Middle-Aged Adults with Type 2 Diabetes
Source: Nutrients. 2023 Jul 3;15(13):3027. doi: 10.3390/nu15133027 (PMC10346630; doi:10.3390/nu15133027)
Supplement: Supplementary file 1 [file nutrients-15-03027-s001.zip › nutrients-2454988-supplementary.pdf]

Effects of endurance exercise and vitamin D supplementation on insulin resistance and plasma lipidome in middle-aged adults with type 2 diabetes

Supplemental tables

Supplementary Table S1 The protocol of endurance exercise training
Supplementary Table S2 Procedures of lipidomic measurement
Supplementary Table S3 Mean differences (95% CI) of blood parameters from the pre (0-week) with each group using 2×2 factorial ANOVA
Supplementary Table S4 Mean differences of glycemic control indicators at three time point with each group
Supplementary Table S5 The 105 lipids that had pronounced changes between t2 and t1 sampling in VD, EX, or EX+VD and their corresponding module colors of WGCNA
Supplementary Table S6 Different co-expression modules of lipids from WGCNA

Table S1. The protocol of endurance exercise training

**Stage**	**HRmax (%)**	**RPE**	**Time**
Week 1	65-70%	11-13	60 min (including 5 min warm up, and 5 min recovery exercise)
Week 2-4	70-75%	11-13	60 min (including 5 min warm up, and 5 min recovery exercise)
Week 5-8	75-80%	12-13	60 min (including 5 min warm up, and 5 min recovery exercise)
Week 9-12	80%	13	60 min (including 5 min warm up, and 5 min recovery exercise)

Note: HRmax, Maximum heart rate; RPE, Rating of perceived exertion.

Table S2. Procedures of lipidomic measurement

**1)**	**Sample preparation**<br>A total of 100 μL plasma samples was properly mixed with 800 μL cold methyl tert-butyl ether, followed by adding 240 μL methanol. The mixture was vortexed for 30 seconds, sonicated at 4°C for 20 min and was centrifuged (14000 g for 15 minutes at 10 °C). The upper organic layer containing lipids was then dried in a vacuum centrifuge for instrumental analyses.
**2)**	**Lipid extraction**<br>The lipid extracts were resuspended in 200 μL isopropanol acetonitrile 9:1 (v/v). Chromatographic separation of 1 μL of the extracted lipids was performed on ultra-high-performance liquid chromatography system LC-30A (Nexera). Analyses were conducted by Shanghai Applied Protein Technology Co., Ltd. In brief, lipids were separated on a Waters ACQUITY PREMIER CSH C18 Column (1.7 μm, 2.1*100 mm), under the following chromatographic conditions: mobile phase A (acetonitrile: water = 6:4, v/v) and mobile phase B (acetonitrile: isopropanol = 1:9, v/v) at a flow rate of 300 μL/min; column oven temperature at 45°C; gradients started with 30% of B for 2 min, then increased to 100% of B over 23 min. The gradient was returned to 30% B over 1 min, and was finally equilibrated for 9 min.
**3)**	**Mass detection**<br>The mass detection was performed using a Thermo Scientific™ Q Exactive mass spectrometer mass spectrometer, equipped with a jet stream electrospray ionization. Data were acquired in both positive and negative ion modes, respectively. Data-dependent acquisition method was used for MS/MS analyses of lipidome. Specifically, 10 MS2 scans were collected after each MS1 full scan. The resolution of MS1 is 70000 at m/z 200. The resolution of MS2 is 17500 at m/z 200. The ESI conditions are: Heater Temp 300 °C, Sheath Gas Flow rate 45 arb, Aux Gas Flow Rate15 arb, Sweep Gas Flow Rate 1arb, spray voltage 3.0KV, Capillary Temp 350 °C, S-Lens RF Level 50%, MS1 scan ranges: 200-1800.
**4)**	**Quality control**<br>The quality control was performed throughout the dataset by including blanks, pure standard samples, extracted standard samples, and pooled plasma samples from this study. Lipidsearch 4.0 software was used for peak detection and lipids annotation. Main parameters are as follows: precursor tolerance 5 ppm,product tolerance 5 ppm, product ion threshold 5%. For the lipid species, fatty acyl chains were abbreviated Cx:y, where x represents the number of carbon atoms and y the number of double bonds of the fatty acyl chain.

Table S3. Mean differences (95% CI) of blood parameters from the pre (0-week) with each group using 2×2 factorial ANOVA

Variables	EX + VD	VD	EX	Con	Vitamin D effect		Exercise effect		Interaction effect	
	N=15	N=15	N=14	N=15	P	Partial η^2	P	Partial η^2	P	Partial η^2
Δ Total body fat (%) [a]	-1.0 (-2.1, 0.2)	0.4 (-0.7, 1.6)	**-1.1 (-2.0, -0.2)**	-0.5 (-1.2, 0.2)	0.262	0.024	**0.032**	0.084	0.428	0.012
Δ Apolipoprotein A (g/L)	0.02 (-0.10, 0.15)	0.03 (-0.07, 0.12)	0.01 (-0.06, 0.07)	0.09 (-0.02, 0.21)	0.582	0.004	0.349	0.009	0.400	0.010
Δ Apolipoprotein B (g/L)	0.02 (-0.07, 0.12)	-0.02 (-0.10, 0.06)	0.03 (-0.06, 0.12)	0.07 (-0.01, 0.14)	0.232	0.020	0.986	0.000	0.327	0.013
Δ Low density lipoprotein (mmol/L)	-0.09 (-0.43, 0.25)	-0.25 (-0.57, 0.06)	-0.26 (-0.54, 0.01)	-0.09 (-0.23, 0.05)	0.969	0.000	0.977	0.000	0.203	0.026
Δ High density lipoprotein (mmol/L)	0.06 (-0.05, 0.17)	0.00 (-0.12, 0.13)	0.01 (-0.12, 0.15)	-0.01 (-0.12, 0.10)	0.563	0.009	0.455	0.018	0.777	0.000
Δ Cholesterol (mmol/L)	0.06 (-0.39, 0.51)	-0.19 (-0.29, 0.29)	-0.19 (-0.50, 0.12)	-0.07 (-0.31, 0.16)	0.699	0.005	0.691	0.004	0.281	0.015
Δ Triglyceride (mmol/L)	-0.04 (-0.26, 0.18)	-0.00 (-0.34, 0.33)	-0.24 (-0.66, 0.18)	0.32 (0.04, 0.59)	0.698	0.015	0.050	0.048	0.085	0.089
Δ HbA1c (%)	-0.8 (-1.6, 0.1)	-0.3 (-1.0, 0.3)	-0.2 (-0.5, 0.2)	-0.5 (-1.3, 0.3)	0.506	0.023	0.820	0.002	0.229	0.014
Δ Fasting blood glucose (mmol/L)	-0.9 (-1.6, -0.2)	-0.9 (-1.8, -0.1)	-0.6 (-1.3, 0.2)	0.4 (-1.8, 1.1)	0.337	0.086	0.884	0.010	0.798	0.033
Δ Fasting insulin (μU/mL)	-1.8 (-6.1, 2.5)	1.3 (-1.3, 4.0)	0.5 (-3.8, 4.8)	0.5 (-3.6, 4.5)	0.806	0.004	0.342	0.023	0.594	0.012
Δ HOMA-IR	-0.9 (-2.4, 0.5)	-0.2 (-1.1, 0.7)	-0.2 (-1.6, 1.3)	0.2 (-1.8, 2.3)	0.447	0.026	0.273	0.027	0.754	0.000
Δ Glucose AUC [b]	-78.1 (-225.8, 69.6)	-140.1 (-307.2, 27.0)	-75.9 (-201.7, 49.9)	36.1 (-112.0, 184.2)	0.197	0.032	0.716	0.003	0.208	0.031
Δ Insulin AUC [b]	515.8 (-1106.7, 2138.2)	1018.1 (-128.1, 2164.3)	1205.9 (-161.9, 2249.9)	-149.1 (-1979.0, 1680.9)	0.724	0.002	0.529	0.008	0.173	0.036

Δ, changes from the endpoint to the pre; EX+VD, vitamin D supplementation and exercise training; VD, vitamin D supplementation; EX, exercise training; Con, control group; HOMA-IR, homeostasis model assessment of insulin resistance; HbA1c, glycated hemoglobin; AUC, area under the curves during oral glucose tolerance test; CI, confidence interval. [a], n=14 in VD and Con; [b], n=13 in VD and Con were included.

Table S4. Mean differences of glycemic control indicators at three time point with each group

Variable	HbA1c (%)	Fasting glucose (mmol/L)	Fasting insulin (μU/mL)	HOMA-IR
EX+VD				
t1	7.0±1.9	6.7±1.4	12.6±8.6	3.9±3.0
t2	6.3±0.7	5.9±1.0	10.8±6.2	3.0±2.2
t3	6.3±0.9	6.2±1.3	10.6±6.0	3.0±2.0
P_{t1-t2}	0.064	**0.016**	0.389	0.086
P_{t2-t3}	0.200	0.123	0.430	0.222
P_{t1-t3}	0.067	0.250	0.737	0.549
EX				
t1	6.7±1.2	7.8±2.1	11.7±6.1	3.9±2.3
t2	6.5±1.1	7.2±2.1	12.1±8.3	3.8±2.3
t3	6.6±1.1	7.6±2.9	10.4±5.3	3.4±1.8
P_{t1-t2}	0.316	0.114	0.809	0.823
P_{t2-t3}	0.083	0.365	0.483	0.288
P_{t1-t3}	0.457	0.722	0.391	0.511
VD				
t1	6.9±1.4	7.4±1.9	9.7±5.6	3.4±2.6
t2	6.6±0.8	6.5±0.8	11.0±8.9	3.2±2.7
t3	6.8±1.1	7.2±1.8	12.7±10.1	4.5±4.6
P_{t1-t2}	0.318	**0.032**	0.294	0.649
P_{t2-t3}	0.471	0.223	0.248	0.183
P_{t1-t3}	0.538	0.217	0.083	0.205
Con				
t1	7.2±1.6	7.5±2.6	14.7±7.6	4.9±2.6
t2	6.7±0.8	7.1±1.3	15.1±11.0	5.1±4.9
t3	6.7±0.6	7.1±1.1	15.8±7.7	5.1±2.9
P_{t1-t2}	0.186	0.588	0.813	0.814
P_{t2-t3}	0.968	0.649	0.993	0.759
P_{t1-t3}	0.354	0.966	0.276	0.432

Data are mean ± standard deviation (SD). EX+VD, vitamin D supplementation and exercise training; VD, vitamin D supplementation; EX, exercise training; Con, control group; HbA1c, glycated hemoglobin; HOMA-IR, homeostasis model assessment of insulin resistance; P_{t1-t2},comparisons between baseline and endpoint with each group were assessed using paired t-test; P_{t2-t3},comparisons between endpoint and flow-up with each group were assessed using paired t-test; P_{t1-t3},comparisons between baseline and flow-up with each group were assessed using paired t-test. t1 and t2: EX+VD, n=15; EX, n=14; VD, n=15; Con, n=15; t3: EX+VD, n=13; EX, n=12; VD, n=11; Con, n=12.

Table S5. The 105 lipids that had pronounced changes between t2 and t1 sampling in VD, EX, or EX+VD and their corresponding module colors of WGCNA

Name	LipidGroup	Class	moduleColors	Con_t1	EX_t1	VD_t1	EX+VD_t1	Con_t2	EX_t2	VD_t2	EX+VD_t2	Con_t3	EX_t3	VD_t3	VD+EX_t3
TG(18:2_18:2_20:4)+Na	TG(56:8)+Na	TG	blue	8.3e+07 ± 6.7e+07	5.1e+07 ± 4.4e+07	6.1e+07 ± 5.3e+07	6.4e+07 ± 6.6e+07	8.0e+07 ± 5.4e+07	5.5e+07 ± 4.8e+07	7.8e+07 ± 8.3e+07	6.9e+07 ± 7.2e+07	6.9e+07 ± 5.2e+07	5.5e+07 ± 3.7e+07	1.1e+08 ± 1.0e+08	7.8e+07 ± 6.5e+07
CerG2GNAc1(t41:5)-2H	CerG2GNAc1(t41:5)-2H	CerG2GNAc1	blue	9.4e+05 ± 5.3e+05	9.6e+05 ± 8.7e+05	9.4e+05 ± 4.3e+05	8.7e+05 ± 3.9e+05	8.8e+05 ± 4.2e+05	7.9e+05 ± 4.7e+05	1.0e+06 ± 5.4e+05	1.0e+06 ± 4.2e+05	7.8e+05 ± 3.2e+05	7.7e+05 ± 5.1e+05	1.0e+06 ± 6.2e+05	1.1e+06 ± 6.7e+05
PE(16:0e_22:6)-H	PE(38:6e)-H	PE	blue	1.3e+07 ± 6.5e+06	8.9e+06 ± 4.4e+06	1.2e+07 ± 5.7e+06	1.0e+07 ± 5.2e+06	1.2e+07 ± 6.9e+06	1.2e+07 ± 9.7e+06	1.3e+07 ± 7.8e+06	1.2e+07 ± 8.0e+06	1.2e+07 ± 5.7e+06	1.0e+07 ± 3.5e+06	1.4e+07 ± 5.5e+06	1.3e+07 ± 6.8e+06
PC(16:1e_16:0)+HCOO	PC(32:1e)+HCOO	PC	blue	2.1e+07 ± 8.1e+06	1.7e+07 ± 9.7e+06	2.0e+07 ± 8.5e+06	1.8e+07 ± 7.4e+06	2.2e+07 ± 9.4e+06	1.5e+07 ± 1.0e+07	2.1e+07 ± 9.5e+06	2.1e+07 ± 9.3e+06	2.0e+07 ± 8.9e+06	1.7e+07 ± 8.0e+06	2.4e+07 ± 9.6e+06	2.3e+07 ± 1.1e+07
PS(40:7e)-H	PS(40:7e)-H	PS	blue	3.9e+07 ± 1.5e+07	3.4e+07 ± 1.7e+07	3.4e+07 ± 1.3e+07	3.4e+07 ± 1.6e+07	3.8e+07 ± 1.6e+07	3.1e+07 ± 1.6e+07	3.6e+07 ± 1.5e+07	3.5e+07 ± 1.4e+07	3.7e+07 ± 1.5e+07	3.4e+07 ± 1.7e+07	3.9e+07 ± 1.4e+07	3.6e+07 ± 1.3e+07
SM(t40:0)+HCOO	SM(t40:0)+HCOO	SM	blue	1.8e+07 ± 6.8e+06	1.7e+07 ± 6.6e+06	1.7e+07 ± 6.7e+06	1.4e+07 ± 5.4e+06	1.8e+07 ± 7.8e+06	1.5e+07 ± 6.5e+06	1.9e+07 ± 5.6e+06	1.7e+07 ± 6.1e+06	1.7e+07 ± 7.1e+06	1.8e+07 ± 9.0e+06	2.0e+07 ± 5.1e+06	1.8e+07 ± 6.1e+06
Hex3Cer(d36:1)+HCOO	Hex3Cer(d36:1)+HCOO	Hex3Cer	blue	8.6e+05 ± 2.9e+05	6.8e+05 ± 2.7e+05	8.2e+05 ± 2.8e+05	7.2e+05 ± 2.6e+05	8.5e+05 ± 3.6e+05	7.0e+05 ± 4.6e+05	9.0e+05 ± 2.8e+05	9.1e+05 ± 4.3e+05	8.2e+05 ± 3.7e+05	6.8e+05 ± 3.1e+05	1.0e+06 ± 3.9e+05	8.9e+05 ± 4.3e+05
SM(d36:2)+H	SM(d36:2)+H	SM	blue	6.4e+08 ±	5.3e+08 ±	5.7e+08 ±	5.4e+08 ±	6.3e+08 ±	5.4e+08 ±	6.3e+08 ±	6.1e+08 ±	5.9e+08 ±	5.4e+08 ±	7.0e+08 ±	6.3e+08 ±

				2.3e+08	1.8e+08	2.2e+08	2.1e+08	2.8e+08	2.3e+08	2.8e+08	2.2e+08	2.4e+08	1.7e+08	2.1e+08	2.6e+08
TG(16:0_16:0_20:4)+Na	TG(52:4)+Na	TG	blue	2.8e+07 ± 2.2e+07	2.5e+07 ± 2.2e+07	1.9e+07 ± 1.4e+07	2.2e+07 ± 2.1e+07	2.8e+07 ± 1.7e+07	3.1e+07 ± 2.2e+07	3.1e+07 ± 3.7e+07	2.6e+07 ± 2.1e+07	2.2e+07 ± 1.9e+07	2.5e+07 ± 2.3e+07	4.4e+07 ± 5.2e+07	2.7e+07 ± 1.9e+07
PC(8:0_11:2)+HCOO	PC(19:2)+HCOO	PC	blue	1.1e+06 ± 4.4e+05	9.9e+05 ± 3.7e+05	1.3e+06 ± 5.1e+05	1.1e+06 ± 4.8e+05	1.0e+06 ± 5.3e+05	1.1e+06 ± 5.9e+05	1.4e+06 ± 5.8e+05	1.3e+06 ± 5.1e+05	9.2e+05 ± 2.9e+05	1.1e+06 ± 4.3e+05	1.3e+06 ± 4.1e+05	1.3e+06 ± 5.4e+05
PC(16:1e_22:5)+HCOO	PC(38:6e)+HCOO	PC	blue	8.2e+06 ± 5.1e+06	6.1e+06 ± 3.4e+06	7.0e+06 ± 3.2e+06	6.5e+06 ± 3.3e+06	8.0e+06 ± 4.4e+06	5.4e+06 ± 4.1e+06	7.9e+06 ± 3.8e+06	6.9e+06 ± 3.5e+06	7.6e+06 ± 4.5e+06	6.6e+06 ± 3.9e+06	7.9e+06 ± 3.0e+06	8.5e+06 ± 4.6e+06
PC(18:1e_22:6)+HCOO	PC(40:7e)+HCOO	PC	blue	3.2e+06 ± 1.6e+06	2.4e+06 ± 1.8e+06	2.8e+06 ± 1.7e+06	2.5e+06 ± 1.8e+06	3.2e+06 ± 2.0e+06	2.4e+06 ± 3.1e+06	2.9e+06 ± 2.0e+06	3.1e+06 ± 2.0e+06	2.8e+06 ± 1.5e+06	3.0e+06 ± 2.1e+06	2.8e+06 ± 2.2e+06	3.1e+06 ± 2.3e+06
ST(t39:1+O)+H	ST(t39:1+O)+H	ST	blue	5.7e+06 ± 1.9e+06	4.7e+06 ± 2.1e+06	5.1e+06 ± 1.6e+06	5.0e+06 ± 2.2e+06	5.5e+06 ± 1.6e+06	4.8e+06 ± 2.2e+06	6.2e+06 ± 4.7e+06	6.0e+06 ± 2.9e+06	5.4e+06 ± 1.6e+06	5.0e+06 ± 1.9e+06	5.8e+06 ± 1.6e+06	6.4e+06 ± 3.5e+06
TG(22:5_18:2_18:2)+Na	TG(58:9)+Na	TG	blue	4.0e+07 ± 3.8e+07	2.0e+07 ± 1.7e+07	3.6e+07 ± 3.8e+07	2.5e+07 ± 1.9e+07	4.0e+07 ± 3.5e+07	2.4e+07 ± 2.1e+07	4.0e+07 ± 4.8e+07	3.0e+07 ± 2.9e+07	3.3e+07 ± 2.2e+07	2.2e+07 ± 1.6e+07	5.7e+07 ± 7.7e+07	3.6e+07 ± 2.7e+07
LPE(18:1e)-H	LPE(18:1e)-H	LPE	blue	3.5e+06 ± 1.8e+06	2.7e+06 ± 1.8e+06	3.5e+06 ± 1.6e+06	2.9e+06 ± 1.3e+06	3.3e+06 ± 1.9e+06	2.8e+06 ± 1.7e+06	3.6e+06 ± 1.4e+06	3.2e+06 ± 1.7e+06	2.6e+06 ± 9.9e+05	2.7e+06 ± 1.3e+06	3.1e+06 ± 1.2e+06	3.8e+06 ± 2.1e+06
LPC(16:1e)+HCOO	LPC(16:1e)+HCOO	LPC	blue	6.7e+06 ±	5.4e+06 ±	8.2e+06 ±	5.8e+06 ±	6.1e+06 ±	6.2e+06 ±	8.5e+06 ±	6.8e+06 ±	5.1e+06 ±	5.6e+06 ±	6.5e+06 ±	7.0e+06 ±

				3.1e+06	2.1e+06	3.4e+06	2.5e+06	2.4e+06	3.6e+06	4.0e+06	2.5e+06	1.6e+06	2.4e+06	2.5e+06	3.3e+06
PE(10:0_11:2)-H	PE(21:2)-H	PE	blue	9.9e+05 ± 4.9e+05	7.4e+05 ± 4.3e+05	1.0e+06 ± 4.4e+05	7.8e+05 ± 3.7e+05	9.1e+05 ± 5.0e+05	8.0e+05 ± 4.6e+05	9.9e+05 ± 3.8e+05	8.8e+05 ± 3.9e+05	7.4e+05 ± 2.6e+05	7.6e+05 ± 3.5e+05	8.7e+05 ± 2.6e+05	1.0e+06 ± 5.1e+05
PI(17:0_20:4)-H	PI(37:4)-H	PI	blue	5.4e+06 ± 2.6e+06	4.4e+06 ± 1.8e+06	5.6e+06 ± 2.7e+06	4.7e+06 ± 2.3e+06	5.5e+06 ± 2.4e+06	4.5e+06 ± 2.8e+06	6.2e+06 ± 4.1e+06	5.2e+06 ± 1.9e+06	4.7e+06 ± 1.5e+06	4.4e+06 ± 2.0e+06	5.2e+06 ± 1.3e+06	5.8e+06 ± 2.1e+06
PIP2(35:2e)-H	PIP2(35:2e)-H	PIP2	blue	2.9e+06 ± 9.9e+05	2.6e+06 ± 8.1e+05	3.0e+06 ± 1.5e+06	2.5e+06 ± 1.0e+06	3.0e+06 ± 1.3e+06	2.8e+06 ± 1.4e+06	2.9e+06 ± 9.4e+05	2.7e+06 ± 9.1e+05	2.5e+06 ± 7.6e+05	2.7e+06 ± 1.1e+06	2.8e+06 ± 8.0e+05	2.9e+06 ± 6.2e+05
CerG2GNAc1(d34:4)+HCOO	CerG2GNAc1(d34:4)+HCOO	CerG2GNAc1	blue	1.8e+07 ± 6.3e+06	1.6e+07 ± 4.8e+06	2.2e+07 ± 7.9e+06	1.6e+07 ± 6.0e+06	1.8e+07 ± 6.7e+06	1.9e+07 ± 7.2e+06	2.0e+07 ± 7.3e+06	1.8e+07 ± 7.4e+06	1.4e+07 ± 5.2e+06	1.7e+07 ± 5.0e+06	1.9e+07 ± 4.9e+06	1.9e+07 ± 6.4e+06
LPE(16:1e)+H	LPE(16:1e)+H	LPE	blue	2.4e+06 ± 9.5e+05	1.9e+06 ± 1.2e+06	2.6e+06 ± 1.3e+06	1.9e+06 ± 6.9e+05	2.3e+06 ± 1.1e+06	2.2e+06 ± 8.7e+05	2.5e+06 ± 9.2e+05	2.1e+06 ± 1.1e+06	2.0e+06 ± 6.8e+05	2.0e+06 ± 8.0e+05	2.2e+06 ± 8.6e+05	2.3e+06 ± 1.1e+06
LPE(18:1e)+H	LPE(18:1e)+H	LPE	blue	3.7e+06 ± 1.8e+06	2.9e+06 ± 1.7e+06	3.7e+06 ± 1.6e+06	3.1e+06 ± 1.6e+06	3.7e+06 ± 2.1e+06	3.1e+06 ± 1.4e+06	3.9e+06 ± 1.7e+06	3.5e+06 ± 2.1e+06	3.0e+06 ± 1.1e+06	2.9e+06 ± 1.5e+06	3.4e+06 ± 1.4e+06	4.1e+06 ± 2.5e+06
PC(18:0e_20:4)+Na	PC(38:4e)+Na	PC	blue	1.4e+08 ± 1.0e+08	9.4e+07 ± 6.2e+07	1.2e+08 ± 7.0e+07	1.1e+08 ± 8.6e+07	1.3e+08 ± 7.9e+07	1.1e+08 ± 1.2e+08	1.2e+08 ± 6.3e+07	1.2e+08 ± 9.8e+07	1.3e+08 ± 8.1e+07	1.0e+08 ± 7.0e+07	1.3e+08 ± 5.4e+07	1.4e+08 ± 1.0e+08
LPC(16:1e)+H	LPC(16:1e)+H	LPC	blue	2.3e+07 ±	1.8e+07 ±	2.8e+07 ±	2.0e+07 ±	2.3e+07 ±	2.1e+07 ±	2.7e+07 ±	2.4e+07 ±	1.8e+07 ±	1.9e+07 ±	2.2e+07 ±	2.6e+07 ±

				1.3e+07	7.5e+06	1.2e+07	1.2e+07	1.2e+07	1.2e+07	1.3e+07	1.1e+07	6.4e+06	6.3e+06	8.1e+06	1.6e+07
LPE(18:1e)+Na	LPE(18:1e)+Na	LPE	blue	1.2e+06 ± 6.8e+05	8.7e+05 ± 6.6e+05	1.2e+06 ± 6.1e+05	9.9e+05 ± 6.0e+05	1.2e+06 ± 7.3e+05	9.0e+05 ± 4.5e+05	1.2e+06 ± 5.9e+05	1.1e+06 ± 7.3e+05	9.4e+05 ± 4.3e+05	8.8e+05 ± 5.4e+05	1.0e+06 ± 5.8e+05	1.3e+06 ± 9.3e+05
PI(18:3_18:2)+Na	PI(36:5)+Na	PI	blue	4.9e+06 ± 1.4e+06	4.4e+06 ± 1.3e+06	4.7e+06 ± 1.7e+06	3.9e+06 ± 1.6e+06	4.8e+06 ± 2.3e+06	4.5e+06 ± 1.4e+06	4.3e+06 ± 1.4e+06	4.3e+06 ± 1.3e+06	4.7e+06 ± 1.4e+06	4.4e+06 ± 1.3e+06	4.8e+06 ± 1.6e+06	5.1e+06 ± 1.8e+06
LPC(18:1e)+Na	LPC(18:1e)+Na	LPC	blue	7.9e+05 ± 7.8e+05	4.1e+05 ± 3.2e+05	8.7e+05 ± 8.6e+05	4.7e+05 ± 3.8e+05	7.3e+05 ± 7.6e+05	4.5e+05 ± 4.5e+05	8.6e+05 ± 9.5e+05	6.5e+05 ± 5.8e+05	4.6e+05 ± 3.0e+05	3.5e+05 ± 2.6e+05	5.6e+05 ± 5.3e+05	7.9e+05 ± 7.6e+05
LPC(18:0)-CH3	LPC(18:0)-CH3	LPC	blue	3.0e+07 ± 1.6e+07	2.6e+07 ± 1.1e+07	3.7e+07 ± 1.8e+07	3.2e+07 ± 1.8e+07	2.9e+07 ± 1.2e+07	3.4e+07 ± 2.4e+07	3.7e+07 ± 1.8e+07	3.4e+07 ± 1.9e+07	2.5e+07 ± 1.6e+07	2.5e+07 ± 1.3e+07	3.1e+07 ± 1.2e+07	3.8e+07 ± 2.1e+07
LPC(18:2e)+HCOO	LPC(18:2e)+HCOO	LPC	blue	6.4e+05 ± 3.8e+05	5.1e+05 ± 2.6e+05	8.6e+05 ± 4.6e+05	6.3e+05 ± 3.2e+05	6.0e+05 ± 2.8e+05	5.7e+05 ± 3.7e+05	8.9e+05 ± 4.8e+05	7.2e+05 ± 4.0e+05	4.6e+05 ± 2.2e+05	4.9e+05 ± 2.6e+05	6.7e+05 ± 2.9e+05	7.0e+05 ± 3.7e+05
PIP(18:3e_22:6)-H	PIP(40:9e)-H	PIP	blue	4.1e+06 ± 1.2e+06	3.6e+06 ± 1.1e+06	4.0e+06 ± 1.2e+06	3.3e+06 ± 1.4e+06	4.0e+06 ± 1.7e+06	3.6e+06 ± 1.5e+06	3.7e+06 ± 1.2e+06	3.8e+06 ± 1.3e+06	3.9e+06 ± 1.5e+06	3.6e+06 ± 1.2e+06	4.3e+06 ± 1.5e+06	4.4e+06 ± 1.6e+06
SM(d36:4)+H	SM(d36:4)+H	SM	blue	3.8e+06 ± 3.4e+06	2.3e+06 ± 1.2e+06	3.1e+06 ± 2.3e+06	2.9e+06 ± 2.2e+06	3.9e+06 ± 3.1e+06	2.6e+06 ± 2.0e+06	3.0e+06 ± 2.4e+06	3.0e+06 ± 2.3e+06	3.6e+06 ± 2.6e+06	1.6e+06 ± 6.4e+05	3.9e+06 ± 2.3e+06	3.4e+06 ± 2.5e+06
PC(14:0_22:6)+HCOO	PC(36:6)+HCOO	PC	blue	7.0e+06 ±	7.0e+06 ±	6.6e+06 ±	5.9e+06 ±	7.0e+06 ±	5.9e+06 ±	7.3e+06 ±	5.3e+06 ±	6.8e+06 ±	5.5e+06 ±	8.6e+06 ±	8.1e+06 ±

				6.0e+06	6.4e+06	4.6e+06	3.8e+06	4.3e+06	4.5e+06	5.7e+06	4.0e+06	5.2e+06	3.8e+06	8.2e+06	6.1e+06
PC(16:0_18:1)+HCOO	PC(34:1)+HCOO	PC	blue	2.3e+08 ± 8.6e+07	1.9e+08 ± 8.3e+07	2.0e+08 ± 7.5e+07	2.1e+08 ± 8.7e+07	2.4e+08 ± 8.5e+07	1.8e+08 ± 8.5e+07	2.4e+08 ± 1.2e+08	1.8e+08 ± 4.8e+07	2.1e+08 ± 7.5e+07	1.9e+08 ± 6.2e+07	2.5e+08 ± 9.8e+07	2.5e+08 ± 1.1e+08
TG(18:0_18:2_20:5)+H	TG(56:7)+H	TG	grey	1.6e+07 ± 1.6e+07	1.0e+07 ± 8.1e+06	1.3e+07 ± 1.2e+07	1.1e+07 ± 1.0e+07	1.6e+07 ± 1.4e+07	1.9e+07 ± 2.5e+07	1.9e+07 ± 2.9e+07	2.2e+07 ± 2.6e+07	1.6e+07 ± 1.6e+07	1.0e+07 ± 8.5e+06	3.4e+07 ± 4.5e+07	1.6e+07 ± 1.4e+07
OAHFA(47:6)-H	OAHFA(47:6)-H	OAHFA	grey	1.6e+06 ± 4.9e+05	1.7e+06 ± 6.0e+05	1.6e+06 ± 6.3e+05	1.5e+06 ± 4.2e+05	1.6e+06 ± 5.0e+05	1.7e+06 ± 8.2e+05	1.7e+06 ± 7.8e+05	1.7e+06 ± 6.6e+05	1.5e+06 ± 2.5e+05	1.6e+06 ± 6.7e+05	1.5e+06 ± 4.1e+05	1.7e+06 ± 7.0e+05
PE(37:3e)+Na	PE(37:3e)+Na	PE	grey	5.8e+07 ± 1.2e+08	1.8e+07 ± 1.2e+07	2.9e+07 ± 2.7e+07	1.9e+07 ± 1.3e+07	3.7e+07 ± 4.6e+07	2.2e+07 ± 3.8e+07	3.4e+07 ± 3.0e+07	2.0e+07 ± 1.4e+07	3.2e+07 ± 3.2e+07	2.5e+07 ± 2.3e+07	3.1e+07 ± 3.0e+07	2.4e+07 ± 1.7e+07
DG(26:6e)+Na	DG(26:6e)+Na	DG	grey	6.4e+05 ± 6.1e+05	6.2e+05 ± 4.8e+05	4.5e+05 ± 3.8e+05	3.9e+05 ± 3.3e+05	5.1e+05 ± 4.1e+05	5.1e+05 ± 4.0e+05	3.6e+05 ± 2.3e+05	2.6e+05 ± 1.6e+05	4.5e+05 ± 3.4e+05	5.1e+05 ± 3.3e+05	6.2e+05 ± 5.5e+05	5.0e+05 ± 3.8e+05
PA(17:0_18:2)-H	PA(35:2)-H	PA	grey	6.1e+06 ± 3.7e+06	2.8e+06 ± 2.0e+06	5.9e+06 ± 3.4e+06	5.4e+06 ± 3.8e+06	6.1e+06 ± 4.0e+06	3.2e+06 ± 3.5e+06	4.3e+06 ± 3.7e+06	4.1e+06 ± 4.5e+06	5.0e+06 ± 4.3e+06	2.8e+06 ± 1.9e+06	5.1e+06 ± 4.2e+06	5.6e+06 ± 6.0e+06
PC(22:4_21:1)+HCOO	PC(43:5)+HCOO	PC	grey	8.6e+06 ± 2.6e+06	7.9e+06 ± 3.9e+06	9.9e+06 ± 4.4e+06	8.2e+06 ± 3.1e+06	8.1e+06 ± 3.6e+06	6.8e+06 ± 2.5e+06	9.0e+06 ± 4.0e+06	7.9e+06 ± 2.3e+06	7.1e+06 ± 1.6e+06	6.8e+06 ± 2.9e+06	8.5e+06 ± 2.7e+06	9.1e+06 ± 3.6e+06
SM(d18:2_16:1)+H	SM(d34:3)+H	SM	grey	3.4e+06 ±	2.6e+06 ±	3.4e+06 ±	2.8e+06 ±	3.4e+06 ±	2.6e+06 ±	2.9e+06 ±	3.2e+06 ±	3.2e+06 ±	2.6e+06 ±	3.2e+06 ±	3.8e+06 ±

				2.0e+06	1.2e+06	1.9e+06	1.4e+06	1.9e+06	1.5e+06	1.5e+06	1.6e+06	2.0e+06	8.2e+05	1.2e+06	2.1e+06
PC(37:6)+H	PC(37:6)+H	PC	grey	3.3e+07 ± 3.0e+07	2.3e+07 ± 2.0e+07	3.3e+07 ± 2.9e+07	2.6e+07 ± 2.0e+07	3.3e+07 ± 2.7e+07	2.7e+07 ± 3.6e+07	3.1e+07 ± 2.3e+07	3.0e+07 ± 2.4e+07	3.0e+07 ± 2.7e+07	2.4e+07 ± 1.8e+07	3.7e+07 ± 3.0e+07	4.2e+07 ± 3.9e+07
LPC(15:0)+H	LPC(15:0)+H	LPC	grey	2.0e+07 ± 1.6e+07	1.7e+07 ± 9.3e+06	2.5e+07 ± 1.6e+07	1.8e+07 ± 1.3e+07	2.0e+07 ± 1.4e+07	2.3e+07 ± 1.6e+07	2.5e+07 ± 1.7e+07	2.0e+07 ± 1.4e+07	1.5e+07 ± 1.2e+07	1.6e+07 ± 8.8e+06	2.1e+07 ± 1.3e+07	2.1e+07 ± 1.5e+07
TG(15:0_18:1_18:3)+Na	TG(51:4)+Na	TG	grey	6.2e+06 ± 4.0e+06	4.3e+06 ± 3.2e+06	5.1e+06 ± 3.9e+06	5.7e+06 ± 3.5e+06	6.2e+06 ± 4.4e+06	5.5e+06 ± 3.9e+06	5.5e+06 ± 4.1e+06	6.4e+06 ± 4.2e+06	5.4e+06 ± 4.0e+06	4.5e+06 ± 2.5e+06	7.0e+06 ± 4.5e+06	7.7e+06 ± 4.4e+06
PC(18:3e_22:0)+HCOO	PC(40:3e)+HCOO	PC	grey	1.7e+07 ± 8.9e+06	1.3e+07 ± 6.3e+06	1.4e+07 ± 5.1e+06	1.4e+07 ± 6.7e+06	1.5e+07 ± 7.4e+06	1.2e+07 ± 6.7e+06	1.5e+07 ± 1.0e+07	1.3e+07 ± 6.1e+06	1.4e+07 ± 6.3e+06	1.0e+07 ± 3.1e+06	1.5e+07 ± 8.3e+06	1.5e+07 ± 6.8e+06
Hex2Cer(m42:7)+HCOO	Hex2Cer(m42:7)+HCOO	Hex2Cer	grey	1.6e+06 ± 5.9e+05	1.4e+06 ± 4.8e+05	1.8e+06 ± 9.8e+05	1.6e+06 ± 5.0e+05	1.5e+06 ± 7.3e+05	1.2e+06 ± 4.2e+05	1.7e+06 ± 8.1e+05	1.4e+06 ± 4.9e+05	1.2e+06 ± 3.4e+05	1.3e+06 ± 4.5e+05	1.4e+06 ± 5.2e+05	1.5e+06 ± 5.1e+05
DG(16:0_20:5)+H	DG(36:5)+H	DG	grey	4.6e+06 ± 2.4e+06	5.7e+06 ± 3.8e+06	4.7e+06 ± 3.8e+06	4.2e+06 ± 3.1e+06	5.4e+06 ± 3.9e+06	5.2e+06 ± 3.0e+06	4.7e+06 ± 3.2e+06	4.1e+06 ± 2.8e+06	2.8e+06 ± 1.8e+06	4.3e+06 ± 3.2e+06	5.4e+06 ± 3.9e+06	4.6e+06 ± 5.7e+06
Cer(d44:5)+H	Cer(d44:5)+H	Cer	grey	8.1e+07 ± 3.6e+07	6.7e+07 ± 2.4e+07	9.0e+07 ± 6.0e+07	6.5e+07 ± 1.8e+07	7.8e+07 ± 3.3e+07	6.4e+07 ± 2.0e+07	8.3e+07 ± 3.5e+07	6.3e+07 ± 2.1e+07	5.7e+07 ± 1.9e+07	6.3e+07 ± 2.9e+07	7.8e+07 ± 2.7e+07	6.9e+07 ± 2.3e+07
AcCa(10:3)+H	AcCa(10:3)+H	AcCa	grey	3.8e+06 ±	2.7e+06 ±	3.9e+06 ±	3.3e+06 ±	3.7e+06 ±	2.2e+06 ±	4.0e+06 ±	2.9e+06 ±	3.1e+06 ±	2.4e+06 ±	3.4e+06 ±	3.2e+06 ±

				2.0e+06	1.7e+06	1.8e+06	1.6e+06	4.1e+06	1.1e+06	1.3e+06	1.4e+06	8.0e+05	1.5e+06	1.5e+06	1.6e+06
GM3(m36:2)-2H	GM3(m36:2)-2H	GM3	turquoise	5.4e+05 ± 4.5e+05	4.0e+05 ± 2.6e+05	6.6e+05 ± 4.2e+05	4.7e+05 ± 2.7e+05	4.9e+05 ± 3.5e+05	6.3e+05 ± 5.2e+05	6.7e+05 ± 2.8e+05	6.0e+05 ± 4.5e+05	4.7e+05 ± 4.4e+05	5.0e+05 ± 5.1e+05	6.6e+05 ± 4.3e+05	7.7e+05 ± 6.9e+05
GM3(m37:4)-2H	GM3(m37:4)-2H	GM3	turquoise	3.3e+06 ± 1.1e+06	3.2e+06 ± 1.2e+06	3.4e+06 ± 1.4e+06	3.1e+06 ± 1.2e+06	3.1e+06 ± 1.0e+06	3.2e+06 ± 1.5e+06	3.9e+06 ± 1.2e+06	3.4e+06 ± 1.5e+06	3.0e+06 ± 1.5e+06	2.9e+06 ± 7.8e+05	3.4e+06 ± 1.0e+06	3.6e+06 ± 1.9e+06
SM(d34:1)+HCOO	SM(d34:1)+HCOO	SM	turquoise	2.2e+07 ± 1.9e+07	1.8e+07 ± 1.6e+07	1.6e+07 ± 4.6e+06	1.6e+07 ± 4.5e+06	2.0e+07 ± 6.4e+06	1.6e+07 ± 1.1e+07	1.8e+07 ± 1.2e+07	1.8e+07 ± 6.4e+06	1.8e+07 ± 6.7e+06	1.4e+07 ± 6.2e+06	1.8e+07 ± 3.1e+06	2.1e+07 ± 5.5e+06
TG(4:0_11:0_18:1)+Na	TG(33:1)+Na	TG	turquoise	1.7e+06 ± 1.2e+06	1.3e+06 ± 9.3e+05	1.5e+06 ± 1.1e+06	8.3e+05 ± 3.5e+05	1.5e+06 ± 1.5e+06	1.4e+06 ± 1.0e+06	2.4e+06 ± 3.0e+06	1.1e+06 ± 6.5e+05	1.3e+06 ± 7.5e+05	1.2e+06 ± 9.8e+05	2.0e+06 ± 1.9e+06	2.1e+06 ± 1.8e+06
WE(8:0_19:3)+H	WE(27:3)+H	WE	turquoise	5.2e+05 ± 2.5e+05	4.3e+05 ± 1.9e+05	4.7e+05 ± 2.1e+05	3.8e+05 ± 1.5e+05	5.2e+05 ± 1.9e+05	4.6e+05 ± 2.0e+05	5.9e+05 ± 2.9e+05	4.6e+05 ± 3.0e+05	4.3e+05 ± 2.1e+05	4.3e+05 ± 1.8e+05	5.9e+05 ± 2.2e+05	5.2e+05 ± 3.2e+05
SM(d42:2)+HCOO	SM(d42:2)+HCOO	SM	turquoise	1.0e+09 ± 3.0e+08	8.2e+08 ± 3.6e+08	1.1e+09 ± 4.8e+08	9.8e+08 ± 3.7e+08	9.8e+08 ± 3.8e+08	7.9e+08 ± 4.0e+08	1.0e+09 ± 4.5e+08	8.8e+08 ± 2.3e+08	8.8e+08 ± 3.0e+08	7.7e+08 ± 2.6e+08	1.1e+09 ± 4.0e+08	1.1e+09 ± 4.1e+08
LPC(22:1)+HCOO	LPC(22:1)+HCOO	LPC	turquoise	6.5e+05 ± 6.2e+05	5.6e+05 ± 8.3e+05	2.0e+06 ± 4.3e+06	7.7e+05 ± 7.0e+05	7.1e+05 ± 7.6e+05	5.5e+05 ± 4.9e+05	1.1e+06 ± 1.5e+06	6.2e+05 ± 3.8e+05	3.9e+05 ± 2.1e+05	5.3e+05 ± 5.7e+05	6.2e+05 ± 5.2e+05	9.7e+05 ± 1.0e+06
SM(d44:2)+HCOO	SM(d44:2)+HCOO	SM	turquoise	8.9e+06 ±	7.3e+06 ±	1.0e+07 ±	8.6e+06 ±	9.0e+06 ±	6.9e+06 ±	9.2e+06 ±	7.4e+06 ±	7.8e+06 ±	7.1e+06 ±	9.9e+06 ±	9.6e+06 ±

				3.7e+06	3.5e+06	5.2e+06	4.3e+06	4.5e+06	4.3e+06	4.4e+06	2.6e+06	3.4e+06	3.2e+06	3.9e+06	4.4e+06
AcCa(24:1)+H	AcCa(24:1)+H	AcCa	turquoise	8.7e+05 ± 6.0e+05	6.5e+05 ± 6.6e+05	1.1e+06 ± 1.0e+06	7.6e+05 ± 4.4e+05	9.2e+05 ± 7.0e+05	5.9e+05 ± 4.2e+05	9.3e+05 ± 6.6e+05	7.1e+05 ± 3.7e+05	7.0e+05 ± 4.2e+05	6.6e+05 ± 5.2e+05	8.4e+05 ± 4.7e+05	1.0e+06 ± 8.5e+05
PE(8:1e_12:2)+Na	PE(20:3e)+Na	PE	turquoise	2.2e+06 ± 9.4e+05	1.9e+06 ± 8.7e+05	2.5e+06 ± 1.3e+06	2.2e+06 ± 1.2e+06	2.2e+06 ± 7.7e+05	2.2e+06 ± 8.1e+05	2.6e+06 ± 1.3e+06	2.3e+06 ± 1.3e+06	1.8e+06 ± 7.0e+05	2.0e+06 ± 8.7e+05	2.4e+06 ± 1.3e+06	2.7e+06 ± 1.5e+06
LPC(20:4e)+H	LPC(20:4e)+H	LPC	turquoise	2.3e+06 ± 1.8e+06	1.7e+06 ± 1.9e+06	2.9e+06 ± 2.7e+06	2.0e+06 ± 1.6e+06	2.2e+06 ± 2.3e+06	3.2e+06 ± 4.9e+06	2.4e+06 ± 1.8e+06	2.4e+06 ± 2.7e+06	2.0e+06 ± 2.2e+06	2.0e+06 ± 3.2e+06	2.3e+06 ± 2.7e+06	2.5e+06 ± 1.9e+06
DGDG(43:10e)+H	DGDG(43:10e)+H	DGDG	turquoise	9.7e+07 ± 5.3e+07	7.6e+07 ± 3.6e+07	1.1e+08 ± 6.6e+07	8.1e+07 ± 4.9e+07	9.1e+07 ± 4.7e+07	9.7e+07 ± 4.5e+07	1.0e+08 ± 5.5e+07	9.3e+07 ± 4.9e+07	6.7e+07 ± 4.3e+07	7.7e+07 ± 4.0e+07	1.0e+08 ± 5.4e+07	9.6e+07 ± 5.9e+07
PC(8:0e_9:0)+Na	PC(17:0e)+Na	PC	turquoise	7.4e+06 ± 6.9e+06	5.9e+06 ± 4.8e+06	8.7e+06 ± 7.1e+06	6.7e+06 ± 5.1e+06	6.5e+06 ± 4.4e+06	7.1e+06 ± 5.3e+06	8.2e+06 ± 4.8e+06	8.4e+06 ± 7.3e+06	5.1e+06 ± 4.9e+06	6.2e+06 ± 4.3e+06	6.9e+06 ± 5.0e+06	7.8e+06 ± 5.9e+06
LPC(17:0)+Na	LPC(17:0)+Na	LPC	turquoise	2.0e+06 ± 1.6e+06	1.5e+06 ± 1.1e+06	2.4e+06 ± 1.7e+06	1.6e+06 ± 1.2e+06	1.9e+06 ± 1.5e+06	1.6e+06 ± 1.1e+06	2.1e+06 ± 1.1e+06	1.9e+06 ± 1.5e+06	1.3e+06 ± 8.7e+05	1.5e+06 ± 1.1e+06	1.9e+06 ± 1.2e+06	1.9e+06 ± 1.5e+06
DGDG(47:10e)+H	DGDG(47:10e)+H	DGDG	turquoise	1.8e+07 ± 2.1e+07	1.3e+07 ± 1.6e+07	2.5e+07 ± 2.5e+07	2.0e+07 ± 2.4e+07	1.8e+07 ± 1.9e+07	2.3e+07 ± 3.1e+07	2.8e+07 ± 2.7e+07	2.3e+07 ± 2.9e+07	1.3e+07 ± 2.1e+07	1.1e+07 ± 1.6e+07	1.8e+07 ± 1.7e+07	3.3e+07 ± 3.3e+07
LPC(20:3e)+H	LPC(20:3e)+H	LPC	turquoise	3.2e+06 ±	2.3e+06 ±	3.7e+06 ±	2.4e+06 ±	3.0e+06 ±	3.8e+06 ±	3.4e+06 ±	3.6e+06 ±	2.9e+06 ±	2.4e+06 ±	3.1e+06 ±	4.3e+06 ±

				2.6e+06	3.0e+06	4.3e+06	3.0e+06	2.4e+06	5.3e+06	3.2e+06	5.1e+06	4.0e+06	4.2e+06	3.2e+06	4.9e+06
LPE(16:0)-H	LPE(16:0)-H	LPE	turquoise	1.9e+07 ± 1.1e+07	1.6e+07 ± 7.3e+06	2.5e+07 ± 1.4e+07	1.9e+07 ± 1.3e+07	1.8e+07 ± 9.2e+06	2.3e+07 ± 1.4e+07	2.7e+07 ± 1.8e+07	2.1e+07 ± 1.4e+07	1.6e+07 ± 1.2e+07	1.8e+07 ± 8.0e+06	2.3e+07 ± 1.3e+07	2.4e+07 ± 1.4e+07
LPC(16:0)-CH3	LPC(16:0)-CH3	LPC	turquoise	8.4e+07 ± 3.4e+07	7.2e+07 ± 2.0e+07	1.0e+08 ± 4.6e+07	7.7e+07 ± 2.9e+07	7.9e+07 ± 2.6e+07	8.8e+07 ± 4.1e+07	1.0e+08 ± 4.6e+07	8.4e+07 ± 3.3e+07	7.0e+07 ± 3.4e+07	7.2e+07 ± 3.0e+07	8.7e+07 ± 3.2e+07	9.0e+07 ± 4.2e+07
LPC(16:0)-CH3	LPC(16:0)-CH3	LPC	turquoise	3.2e+06 ± 1.4e+06	2.8e+06 ± 1.1e+06	4.0e+06 ± 1.7e+06	3.2e+06 ± 1.4e+06	3.1e+06 ± 1.1e+06	3.4e+06 ± 1.5e+06	4.4e+06 ± 2.5e+06	3.6e+06 ± 1.9e+06	2.8e+06 ± 1.4e+06	2.7e+06 ± 9.7e+05	3.6e+06 ± 1.3e+06	4.2e+06 ± 2.1e+06
LPC(17:0)-CH3	LPC(17:0)-CH3	LPC	turquoise	1.0e+06 ± 7.5e+05	8.0e+05 ± 5.6e+05	1.3e+06 ± 7.9e+05	9.3e+05 ± 6.1e+05	9.4e+05 ± 6.4e+05	1.0e+06 ± 7.1e+05	1.2e+06 ± 6.7e+05	1.1e+06 ± 9.0e+05	7.6e+05 ± 7.2e+05	8.1e+05 ± 5.4e+05	8.5e+05 ± 5.7e+05	1.1e+06 ± 7.8e+05
LPC(18:1)-CH3	LPC(18:1)-CH3	LPC	turquoise	1.5e+07 ± 7.2e+06	1.3e+07 ± 6.7e+06	2.1e+07 ± 1.2e+07	1.4e+07 ± 7.2e+06	1.5e+07 ± 5.8e+06	1.7e+07 ± 1.1e+07	2.1e+07 ± 1.2e+07	1.5e+07 ± 7.3e+06	1.4e+07 ± 8.2e+06	1.2e+07 ± 7.0e+06	1.6e+07 ± 6.3e+06	1.7e+07 ± 8.2e+06
LPC(16:1)+HCOO	LPC(16:1)+HCOO	LPC	turquoise	1.8e+07 ± 1.2e+07	1.5e+07 ± 4.5e+06	2.1e+07 ± 8.6e+06	1.5e+07 ± 5.7e+06	1.9e+07 ± 1.2e+07	2.1e+07 ± 1.3e+07	2.3e+07 ± 1.1e+07	1.7e+07 ± 8.2e+06	1.6e+07 ± 9.0e+06	1.6e+07 ± 7.1e+06	1.9e+07 ± 7.2e+06	1.6e+07 ± 6.7e+06
LPC(16:0)+HCOO	LPC(16:0)+HCOO	LPC	turquoise	1.1e+09 ± 4.4e+08	9.8e+08 ± 2.7e+08	1.3e+09 ± 4.9e+08	9.9e+08 ± 3.4e+08	1.1e+09 ± 3.2e+08	1.2e+09 ± 6.1e+08	1.3e+09 ± 5.7e+08	1.1e+09 ± 4.9e+08	9.2e+08 ± 3.7e+08	1.0e+09 ± 3.8e+08	1.2e+09 ± 3.4e+08	1.2e+09 ± 4.7e+08
LPC(19:0)+H	LPC(19:0)+H	LPC	turquoise	5.8e+06 ±	4.4e+06 ±	6.8e+06 ±	4.8e+06 ±	5.4e+06 ±	5.9e+06 ±	6.3e+06 ±	5.8e+06 ±	4.8e+06 ±	4.5e+06 ±	5.9e+06 ±	7.5e+06 ±

				4.5e+06	3.2e+06	4.2e+06	2.7e+06	3.0e+06	5.4e+06	3.9e+06	4.1e+06	3.3e+06	3.5e+06	2.7e+06	5.2e+06
LPC(17:0)+HCOO	LPC(17:0)+HCOO	LPC	turquoise	1.4e+07 ± 9.5e+06	1.0e+07 ± 6.1e+06	1.7e+07 ± 1.2e+07	1.2e+07 ± 7.0e+06	1.2e+07 ± 6.4e+06	1.3e+07 ± 9.5e+06	1.6e+07 ± 9.9e+06	1.4e+07 ± 9.0e+06	1.0e+07 ± 9.3e+06	1.2e+07 ± 7.8e+06	1.5e+07 ± 1.2e+07	1.4e+07 ± 8.5e+06
LPC(18:1)+HCOO	LPC(18:1)+HCOO	LPC	turquoise	2.3e+08 ± 1.0e+08	2.1e+08 ± 1.2e+08	3.3e+08 ± 1.9e+08	2.2e+08 ± 9.5e+07	2.3e+08 ± 8.4e+07	2.7e+08 ± 1.9e+08	2.9e+08 ± 1.1e+08	2.3e+08 ± 1.1e+08	2.1e+08 ± 1.1e+08	2.1e+08 ± 1.3e+08	2.6e+08 ± 1.2e+08	2.9e+08 ± 1.6e+08
LPC(18:0)+HCOO	LPC(18:0)+HCOO	LPC	turquoise	4.4e+08 ± 2.3e+08	4.1e+08 ± 1.6e+08	5.5e+08 ± 2.5e+08	4.4e+08 ± 2.3e+08	4.4e+08 ± 1.7e+08	5.5e+08 ± 4.1e+08	5.6e+08 ± 2.6e+08	5.2e+08 ± 3.2e+08	3.8e+08 ± 2.4e+08	3.9e+08 ± 1.9e+08	4.9e+08 ± 1.8e+08	5.8e+08 ± 3.1e+08
LPC(19:0)+HCOO	LPC(19:0)+HCOO	LPC	turquoise	1.5e+06 ± 9.1e+05	1.3e+06 ± 8.0e+05	1.8e+06 ± 9.6e+05	1.3e+06 ± 6.1e+05	1.3e+06 ± 6.0e+05	1.6e+06 ± 1.4e+06	1.7e+06 ± 7.2e+05	1.6e+06 ± 9.1e+05	1.2e+06 ± 7.7e+05	1.3e+06 ± 8.7e+05	1.5e+06 ± 6.2e+05	1.8e+06 ± 9.7e+05
PC(10:0e_8:0)+Na	PC(18:0e)+Na	PC	turquoise	1.2e+07 ± 9.3e+06	7.7e+06 ± 5.5e+06	1.6e+07 ± 1.3e+07	1.1e+07 ± 9.1e+06	1.2e+07 ± 8.3e+06	1.1e+07 ± 9.1e+06	1.5e+07 ± 1.2e+07	1.6e+07 ± 2.3e+07	8.7e+06 ± 6.6e+06	8.3e+06 ± 8.7e+06	1.1e+07 ± 8.6e+06	1.8e+07 ± 1.7e+07
PC(8:0e_12:2)+H	PC(20:2e)+H	PC	turquoise	1.5e+07 ± 1.9e+07	1.0e+07 ± 7.2e+06	2.0e+07 ± 1.8e+07	1.3e+07 ± 1.3e+07	1.3e+07 ± 7.6e+06	1.7e+07 ± 1.8e+07	1.8e+07 ± 1.5e+07	2.2e+07 ± 4.5e+07	1.2e+07 ± 9.9e+06	1.1e+07 ± 1.3e+07	2.0e+07 ± 2.8e+07	1.7e+07 ± 1.7e+07
LPC(20:0)+H	LPC(20:0)+H	LPC	turquoise	8.0e+06 ± 4.9e+06	6.4e+06 ± 3.9e+06	1.0e+07 ± 6.3e+06	8.2e+06 ± 5.0e+06	7.8e+06 ± 4.2e+06	8.3e+06 ± 7.0e+06	8.6e+06 ± 3.8e+06	1.1e+07 ± 1.1e+07	7.7e+06 ± 4.7e+06	6.4e+06 ± 3.6e+06	1.0e+07 ± 4.8e+06	1.1e+07 ± 6.5e+06
CerG2GNAc1(d32:1)-H	CerG2GNAc1(d32:1)-H	CerG2GNAc1	turquoise	3.9e+07 ±	3.4e+07 ±	5.3e+07 ±	3.4e+07 ±	4.0e+07 ±	4.9e+07 ±	5.1e+07 ±	4.1e+07 ±	3.0e+07 ±	3.6e+07 ±	4.3e+07 ±	4.4e+07 ±

				2.1e+07	1.4e+07	2.8e+07	1.8e+07	2.0e+07	3.2e+07	2.9e+07	2.6e+07	1.9e+07	2.0e+07	1.7e+07	2.5e+07
CerG2GNAc1(d36:3)-H	CerG2GNAc1(d36:3)-H	CerG2GNAc1	turquoise	2.0e+06 ± 1.9e+06	1.8e+06 ± 2.9e+06	3.4e+06 ± 2.7e+06	1.7e+06 ± 1.6e+06	1.9e+06 ± 1.3e+06	3.2e+06 ± 4.5e+06	2.8e+06 ± 1.7e+06	2.0e+06 ± 2.0e+06	1.7e+06 ± 2.1e+06	1.8e+06 ± 2.7e+06	2.6e+06 ± 2.6e+06	2.3e+06 ± 1.6e+06
CerG2GNAc1(d36:1)-H	CerG2GNAc1(d36:1)-H	CerG2GNAc1	turquoise	8.7e+06 ± 8.4e+06	7.6e+06 ± 6.0e+06	1.3e+07 ± 1.1e+07	9.2e+06 ± 8.9e+06	8.7e+06 ± 5.6e+06	1.5e+07 ± 2.0e+07	1.3e+07 ± 9.6e+06	1.2e+07 ± 1.3e+07	7.4e+06 ± 9.9e+06	7.7e+06 ± 8.0e+06	9.9e+06 ± 6.6e+06	1.4e+07 ± 1.4e+07
GM3(m36:5)-H	GM3(m36:5)-H	GM3	turquoise	8.9e+05 ± 6.7e+05	8.2e+05 ± 1.1e+06	1.5e+06 ± 1.0e+06	8.0e+05 ± 6.1e+05	9.0e+05 ± 5.8e+05	1.3e+06 ± 1.4e+06	1.4e+06 ± 7.4e+05	9.2e+05 ± 7.6e+05	7.5e+05 ± 7.4e+05	8.7e+05 ± 1.0e+06	1.2e+06 ± 1.0e+06	1.0e+06 ± 6.6e+05
CerG2GNAc1(d38:4)+HCOO	CerG2GNAc1(d38:4)+HCOO	CerG2GNAc1	turquoise	5.7e+06 ± 4.7e+06	5.2e+06 ± 3.5e+06	8.4e+06 ± 6.0e+06	5.3e+06 ± 3.2e+06	5.7e+06 ± 3.4e+06	8.1e+06 ± 8.5e+06	7.8e+06 ± 4.6e+06	7.1e+06 ± 6.2e+06	4.6e+06 ± 4.9e+06	5.0e+06 ± 4.4e+06	6.5e+06 ± 3.5e+06	8.6e+06 ± 7.1e+06
GM3(m38:6)+HCOO	GM3(m38:6)+HCOO	GM3	turquoise	3.9e+06 ± 2.6e+06	3.5e+06 ± 1.7e+06	5.2e+06 ± 2.6e+06	3.6e+06 ± 1.8e+06	3.8e+06 ± 2.1e+06	5.0e+06 ± 4.4e+06	5.0e+06 ± 2.3e+06	4.5e+06 ± 3.3e+06	2.9e+06 ± 1.7e+06	3.6e+06 ± 2.3e+06	3.9e+06 ± 1.1e+06	5.2e+06 ± 2.7e+06
GM3(d42:2)-H	GM3(d42:2)-H	GM3	turquoise	5.6e+06 ± 1.8e+06	4.3e+06 ± 2.4e+06	7.3e+06 ± 5.2e+06	5.0e+06 ± 1.8e+06	5.4e+06 ± 2.7e+06	4.6e+06 ± 2.5e+06	6.1e+06 ± 3.2e+06	5.4e+06 ± 2.2e+06	4.4e+06 ± 2.4e+06	4.5e+06 ± 2.6e+06	5.6e+06 ± 1.9e+06	5.9e+06 ± 2.5e+06
PC(31:0)+H	PC(31:0)+H	PC	turquoise	3.0e+07 ± 1.8e+07	2.5e+07 ± 1.4e+07	3.0e+07 ± 1.8e+07	2.6e+07 ± 1.5e+07	3.1e+07 ± 1.5e+07	2.7e+07 ± 1.8e+07	3.0e+07 ± 1.9e+07	2.9e+07 ± 1.4e+07	2.8e+07 ± 1.7e+07	2.5e+07 ± 1.0e+07	3.5e+07 ± 1.1e+07	3.1e+07 ± 2.1e+07
SM(d17:0_21:6)+H	SM(d38:6)+H	SM	turquoise	9.7e+06 ±	7.1e+06 ±	1.1e+07 ±	8.4e+06 ±	9.9e+06 ±	8.0e+06 ±	8.1e+06 ±	1.0e+07 ±	8.4e+06 ±	7.4e+06 ±	1.1e+07 ±	1.0e+07 ±

				6.1e+06	3.4e+06	8.5e+06	5.3e+06	6.0e+06	6.0e+06	3.3e+06	4.9e+06	4.9e+06	2.4e+06	4.9e+06	5.6e+06
LPE(18:2)+H	LPE(18:2)+H	LPE	turquoise	4.5e+07 ± 2.9e+07	4.9e+07 ± 4.8e+07	5.6e+07 ± 2.6e+07	4.3e+07 ± 4.1e+07	4.1e+07 ± 1.5e+07	7.0e+07 ± 7.1e+07	5.1e+07 ± 2.2e+07	4.9e+07 ± 5.9e+07	4.0e+07 ± 2.2e+07	4.7e+07 ± 4.1e+07	6.4e+07 ± 3.6e+07	6.6e+07 ± 6.7e+07
LPC(16:2e)+H	LPC(16:2e)+H	LPC	turquoise	8.8e+06 ± 4.1e+06	7.9e+06 ± 4.1e+06	1.1e+07 ± 6.0e+06	8.5e+06 ± 5.8e+06	9.1e+06 ± 6.8e+06	1.1e+07 ± 8.9e+06	1.1e+07 ± 6.0e+06	9.5e+06 ± 4.6e+06	7.7e+06 ± 6.4e+06	8.2e+06 ± 4.3e+06	9.7e+06 ± 3.8e+06	1.0e+07 ± 7.0e+06
LPC(16:0)+H	LPC(16:0)+H	LPC	turquoise	5.0e+09 ± 2.8e+09	4.0e+09 ± 1.7e+09	5.7e+09 ± 3.2e+09	4.3e+09 ± 2.8e+09	4.9e+09 ± 2.5e+09	5.6e+09 ± 3.9e+09	6.2e+09 ± 4.4e+09	4.8e+09 ± 2.7e+09	3.9e+09 ± 2.6e+09	4.2e+09 ± 2.4e+09	5.3e+09 ± 2.6e+09	5.2e+09 ± 3.1e+09
PC(8:0e_10:0)+H	PC(18:0e)+H	PC	turquoise	1.5e+09 ± 1.0e+09	1.3e+09 ± 7.1e+08	1.8e+09 ± 1.0e+09	1.5e+09 ± 1.0e+09	1.6e+09 ± 8.6e+08	1.9e+09 ± 1.7e+09	1.9e+09 ± 1.1e+09	1.8e+09 ± 1.5e+09	1.3e+09 ± 9.7e+08	1.2e+09 ± 7.4e+08	1.6e+09 ± 8.5e+08	2.0e+09 ± 1.4e+09
WE(16:0)-H	WE(16:0)-H	WE	turquoise	4.1e+06 ± 1.6e+06	3.5e+06 ± 1.7e+06	4.8e+06 ± 2.2e+06	4.0e+06 ± 1.7e+06	4.0e+06 ± 1.6e+06	4.1e+06 ± 1.9e+06	5.0e+06 ± 2.2e+06	4.5e+06 ± 2.1e+06	3.6e+06 ± 1.0e+06	3.3e+06 ± 1.2e+06	3.6e+06 ± 1.5e+06	4.3e+06 ± 2.1e+06
LPC(18:2)-CH3	LPC(18:2)-CH3	LPC	turquoise	2.9e+07 ± 1.9e+07	2.7e+07 ± 2.2e+07	4.0e+07 ± 3.0e+07	2.6e+07 ± 2.1e+07	2.5e+07 ± 9.6e+06	3.3e+07 ± 2.5e+07	3.3e+07 ± 1.3e+07	3.0e+07 ± 2.8e+07	2.3e+07 ± 1.2e+07	2.5e+07 ± 1.9e+07	3.4e+07 ± 1.4e+07	3.4e+07 ± 2.7e+07
LPC(18:0)-CH3	LPC(18:0)-CH3	LPC	turquoise	2.0e+06 ± 7.8e+05	1.9e+06 ± 6.3e+05	2.6e+06 ± 1.1e+06	2.1e+06 ± 9.0e+05	2.1e+06 ± 9.0e+05	2.3e+06 ± 1.4e+06	2.8e+06 ± 1.2e+06	2.5e+06 ± 1.1e+06	1.7e+06 ± 7.6e+05	1.7e+06 ± 4.9e+05	2.2e+06 ± 7.3e+05	3.0e+06 ± 2.0e+06
GM3(m38:2)-2H	GM3(m38:2)-2H	GM3	turquoise	4.5e+06 ±	3.9e+06 ±	7.0e+06 ±	4.4e+06 ±	4.3e+06 ±	5.1e+06 ±	5.9e+06 ±	4.6e+06 ±	3.6e+06 ±	3.6e+06 ±	4.6e+06 ±	5.5e+06 ±

				2.5e+06	3.6e+06	5.5e+06	2.1e+06	2.0e+06	4.5e+06	3.4e+06	2.3e+06	2.5e+06	2.8e+06	2.9e+06	3.3e+06
PC(8:1e_10:2)+Na	PC(18:3e)+Na	PC	turquoise	2.7e+06 ± 2.6e+06	2.7e+06 ± 2.8e+06	3.9e+06 ± 3.9e+06	2.5e+06 ± 1.9e+06	2.6e+06 ± 1.9e+06	4.5e+06 ± 6.3e+06	3.8e+06 ± 3.7e+06	2.8e+06 ± 4.3e+06	2.4e+06 ± 1.9e+06	2.2e+06 ± 2.1e+06	2.6e+06 ± 2.1e+06	3.7e+06 ± 5.0e+06
LPC(20:5)+H	LPC(20:5)+H	LPC	turquoise	2.8e+08 ± 1.4e+08	2.4e+08 ± 1.5e+08	3.8e+08 ± 2.3e+08	2.5e+08 ± 1.3e+08	2.7e+08 ± 1.2e+08	3.3e+08 ± 2.1e+08	3.6e+08 ± 1.7e+08	2.9e+08 ± 1.8e+08	2.5e+08 ± 1.0e+08	2.2e+08 ± 1.1e+08	4.0e+08 ± 2.3e+08	3.3e+08 ± 2.0e+08
CerG3GNAc1(t37:6)-2H	CerG3GNAc1(t37:6)-2H	CerG3GNAc1	turquoise	7.7e+07 ± 3.2e+07	7.5e+07 ± 2.6e+07	9.5e+07 ± 3.6e+07	7.8e+07 ± 3.1e+07	7.8e+07 ± 2.6e+07	8.9e+07 ± 4.5e+07	9.0e+07 ± 2.6e+07	8.8e+07 ± 3.7e+07	6.9e+07 ± 3.4e+07	7.3e+07 ± 2.9e+07	8.7e+07 ± 2.4e+07	9.4e+07 ± 3.7e+07
LPC(18:0)+Na	LPC(18:0)+Na	LPC	turquoise	2.8e+08 ± 2.0e+08	2.3e+08 ± 1.3e+08	3.4e+08 ± 2.1e+08	2.9e+08 ± 1.9e+08	2.9e+08 ± 1.6e+08	3.1e+08 ± 2.2e+08	3.3e+08 ± 1.7e+08	3.2e+08 ± 2.3e+08	2.4e+08 ± 1.8e+08	2.1e+08 ± 1.3e+08	2.9e+08 ± 1.6e+08	3.8e+08 ± 2.5e+08
PC(10:0e_10:1)+H	PC(20:1e)+H	PC	turquoise	1.4e+07 ± 8.9e+06	1.1e+07 ± 1.2e+07	2.3e+07 ± 1.8e+07	1.3e+07 ± 7.4e+06	1.3e+07 ± 6.7e+06	1.5e+07 ± 1.6e+07	1.9e+07 ± 1.3e+07	1.5e+07 ± 9.9e+06	1.2e+07 ± 9.6e+06	1.1e+07 ± 8.8e+06	1.5e+07 ± 1.0e+07	1.8e+07 ± 1.2e+07
LPC(19:0)+Na	LPC(19:0)+Na	LPC	turquoise	1.7e+06 ± 1.6e+06	1.1e+06 ± 1.0e+06	2.3e+06 ± 2.4e+06	1.3e+06 ± 8.4e+05	1.6e+06 ± 1.3e+06	1.5e+06 ± 1.6e+06	1.9e+06 ± 1.5e+06	1.7e+06 ± 1.8e+06	1.2e+06 ± 9.2e+05	9.9e+05 ± 8.5e+05	1.5e+06 ± 1.1e+06	2.6e+06 ± 2.7e+06
CerG2GNAc1(d36:5)+HCOO	CerG2GNAc1(d36:5)+HCOO	CerG2GNAc1	turquoise	4.9e+06 ± 2.6e+06	4.6e+06 ± 3.9e+06	6.6e+06 ± 3.4e+06	4.0e+06 ± 2.1e+06	5.0e+06 ± 3.3e+06	5.3e+06 ± 3.2e+06	6.7e+06 ± 3.8e+06	4.5e+06 ± 2.1e+06	4.5e+06 ± 3.9e+06	4.2e+06 ± 2.3e+06	5.7e+06 ± 2.5e+06	5.2e+06 ± 2.3e+06
GM3(d40:2)-H	GM3(d40:2)-H	GM3	turquoise	1.4e+06 ±	1.2e+06 ±	1.5e+06 ±	1.4e+06 ±	1.4e+06 ±	1.4e+06 ±	1.5e+06 ±	1.6e+06 ±	1.3e+06 ±	1.1e+06 ±	1.7e+06 ±	1.7e+06 ±

LPC(16:0)+Na	LPC(16:0)+Na	LPC	turquoise	5.5e+05 6.2e+08 ± 2.2e+08	5.9e+05 5.7e+08 ± 1.6e+08	8.3e+05 6.8e+08 ± 2.4e+08	5.8e+05 5.8e+08 ± 1.9e+08	7.3e+05 6.1e+08 ± 1.8e+08	9.3e+05 6.4e+08 ± 1.7e+08	8.6e+05 6.2e+08 ± 1.8e+08	7.6e+05 6.5e+08 ± 2.1e+08	8.6e+05 5.1e+08 ± 1.5e+08	4.7e+05 5.6e+08 ± 1.6e+08	7.6e+05 6.5e+08 ± 1.9e+08	8.6e+05 6.3e+08 ± 2.3e+08

Metabolites after log transformation are presented as mean ± SD. WGCNA, weighted gene correlation network analysis; EX+VD, vitamin D supplementation and exercise training; VD, vitamin D supplementation; EX, exercise training; Con, control group; t1, time 1 (0-week); t2, time 2(12-week); t3, time 3 (24-week); TG, triglycerols; PC, phosphatidylcholine; Cer, ceramide; SM, sphingomyelin; PE, phosphatidylethanolamine; LPC, lyso-phosphatidylcholine; DG, diacylglycerols; PS, phosphatidylserine; AcCa, acyl carnitine; HexCer/Hex2Cer, hexosyl ceramide; GM3, ganglioside, monosialo trihexosyl ceramide; PIP, phosphatidylinositol(4)phosphate; CerG3GNAc1, dihexosyl N-acetylhexosyl ceramide; WE, wax exters; PIP2, phosphatidylinositol(4,5)bisphosphate; DGDG, digalactosyl diglyceride.

Table S6. Different co-expression modules of lipids from WGCNA

ID	Time	Group	MEturquoise	MEblue	MEgrey
NO.9	t2	Con	-0.14902	-0.21164	-0.10501
NO.11	t2	Con	-0.12497	-0.17163	0.044051
NO.7	t2	Con	-0.08231	-0.1705	-0.07593
NO.64	t2	Con	-0.14548	-0.12775	0.120497
NO.14	t2	Con	-0.07593	-0.09396	-0.05323
NO.57	t2	Con	-0.03385	-0.06944	-0.05637
NO.42	t2	Con	0.01436	-0.00806	-0.00889
NO.36	t2	Con	0.001078	0.000352	0.220759
NO.38	t2	Con	-0.00037	0.005865	0.102853
NO.41	t2	Con	-0.00819	0.025145	-0.15752
NO.20	t2	Con	0.055918	0.038414	0.051209
NO.34	t2	Con	-0.08257	0.050942	-0.02604
NO.59	t2	Con	0.039487	0.056244	0.115969
NO.39	t2	Con	0.078848	0.151449	-0.08364
NO.56	t2	Con	0.058312	0.153096	0.071819
NO.15	t2	EX	0.214057	-0.33127	-0.22934
NO.5	t2	EX	-0.12099	-0.14307	-0.20828
NO.61	t2	EX	0.624663	-0.09745	-0.05146
NO.4	t2	EX	0.002245	-0.08219	0.030735
NO.52	t2	EX	0.01043	-0.08124	-0.01524
NO.40	t2	EX	-0.02237	-0.03985	-0.0574
NO.63	t2	EX	-0.08475	-0.00143	-0.26457
NO.18	t2	EX	0.052397	0.00438	0.021825
NO.48	t2	EX	0.050366	0.009003	-0.02017
NO.27	t2	EX	0.029604	0.014806	0.053752
NO.13	t2	EX	0.04413	0.05796	0.144222
NO.49	t2	EX	0.02013	0.07619	0.01099
NO.25	t2	EX	0.167324	0.203141	0.270766
NO.43	t2	EX	0.27969	0.309746	-0.06524
NO.16	t2	VD	-0.25667	-0.13338	-0.2095
NO.54	t2	VD	-0.29076	0.157854	0.049957
NO.50	t2	VD	-0.05998	-0.11207	0.129471
NO.33	t2	VD	-0.12463	-0.09882	0.122718
NO.60	t2	VD	-0.05812	-0.0665	-0.04066
NO.62	t2	VD	-0.14805	-0.05179	0.365859
NO.1	t2	VD	0.01411	0.0027	-0.11476
NO.45	t2	VD	0.031488	0.084735	0.013738
NO.47	t2	VD	0.029508	0.089646	-0.11039
NO.26	t2	VD	-0.01676	0.125403	0.225281
NO.17	t2	VD	0.045495	0.11715	-0.14742
NO.22	t2	VD	-0.03488	0.121818	0.126183
NO.6	t2	VD	0.103333	0.136579	-0.01837
NO.19	t2	VD	0.093501	0.172567	0.01401
NO.31	t2	VD	0.146504	0.219633	-0.36501
NO.28	t2	EX+VD	-0.09457	-0.10412	-0.03346
NO.29	t2	EX+VD	0.018104	0.03054	-0.05845
NO.10	t2	EX+VD	-0.06507	0.028361	0.015147

NO.2	t2	EX+VD	-0.01957	0.005179	-0.04044
NO.58	t2	EX+VD	-0.01884	0.003571	0.208005
NO.55	t2	EX+VD	0.008954	0.000775	-0.12782
NO.21	t2	EX+VD	0.001452	0.005407	-0.0147
NO.12	t2	EX+VD	0.085247	0.132154	0.103314
NO.51	t2	EX+VD	0.011978	0.05062	0.095735
NO.24	t2	EX+VD	0.100242	0.064908	0.057968
NO.65	t2	EX+VD	0.204589	0.181325	0.0036
NO.53	t2	EX+VD	0.102646	0.187004	-0.02311
NO.23	t2	EX+VD	0.04235	0.196406	-0.11416
NO.32	t2	EX+VD	0.195223	0.2017	0.086285
NO.44	t2	EX+VD	0.124677	0.216248	0.019867

WGCNA, weighted gene correlation network analysis; EX+VD, vitamin D supplementation and exercise training; VD, vitamin D supplementation; EX, exercise training; Con, control group; t1, time 1 (0-week); t2, time 2 (12-week).
