# Supplementary material for: Effects of Endurance Exercise and Vitamin D Supplementation on Insulin Resistance and Plasma Lipidome in Middle-Aged Adults with Type 2 Diabetes
Source: Nutrients. 2023 Jul 3;15(13):3027. doi: 10.3390/nu15133027 (PMC10346630; doi:10.3390/nu15133027)
Supplement: Supplementary file 1 [file nutrients-15-03027-s001.zip › nutrients-2454988-supplementary.pptx]

## Slide 1
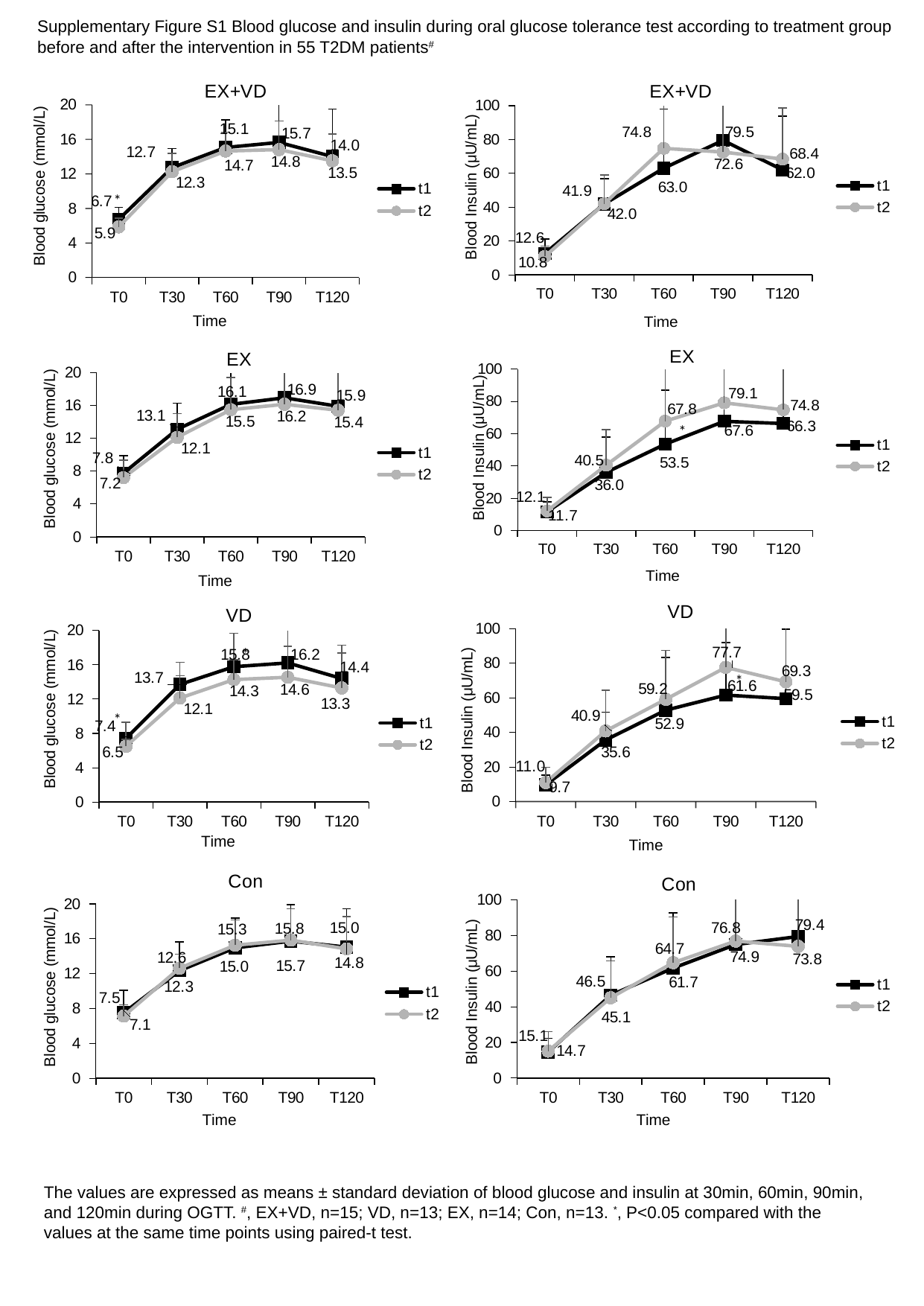

Supplementary Figure S1 Blood glucose and insulin during oral glucose tolerance test according to treatment group before and after the intervention in 55 T2DM patients#
### Chart: EX+VD
| Category | t1 | t2 |
|---|---|---|
| T0 | 6.73 | 5.86 |
| T30 | 12.73 | 12.26 |
| T60 | 15.06 | 14.65 |
| T90 | 15.65 | 14.82 |
| T120 | 14.01 | 13.52 |
### Chart: EX+VD
| Category | t1 | t2 |
|---|---|---|
| T0 | 12.61 | 10.82 |
| T30 | 41.87 | 42.02 |
| T60 | 63.01 | 74.76 |
| T90 | 79.51 | 72.58 |
| T120 | 62.03 | 68.42 |*
### Chart: EX
| Category | t1 | t2 |
|---|---|---|
| T0 | 11.66 | 12.14 |
| T30 | 35.99 | 40.47 |
| T60 | 53.51 | 67.75 |
| T90 | 67.63 | 79.13 |
| T120 | 66.29 | 74.75 |
### Chart: EX
| Category | t1 | t2 |
|---|---|---|
| T0 | 7.77 | 7.222 |
| T30 | 13.1 | 12.1235714285714 |
| T60 | 16.14 | 15.49 |
| T90 | 16.94 | 16.15 |
| T120 | 15.9 | 15.42 |*
### Chart: VD
| Category | t1 | t2 |
|---|---|---|
| T0 | 9.67 | 11.0 |
| T30 | 35.64 | 40.88 |
| T60 | 52.8886666666667 | 59.1753333333333 |
| T90 | 61.6030769230769 | 77.6576923076923 |
| T120 | 59.5313333333333 | 69.286 |
### Chart: VD
| Category | t1 | t2 |
|---|---|---|
| T0 | 7.42 | 6.5 |
| T30 | 13.7092307692308 | 12.1238461538462 |
| T60 | 15.78 | 14.28 |
| T90 | 16.22 | 14.55 |
| T120 | 14.4 | 13.3 |*
*
*
### Chart: Con
| Category | t1 | t2 |
|---|---|---|
| T0 | 7.51 | 7.135 |
| T30 | 12.34 | 12.62 |
| T60 | 14.96 | 15.26 |
| T90 | 15.73 | 15.84 |
| T120 | 15.04 | 14.84 |
### Chart: Con
| Category | t1 | t2 |
|---|---|---|
| T0 | 14.66 | 15.12 |
| T30 | 46.5 | 45.05 |
| T60 | 61.7 | 64.697 |
| T90 | 74.86 | 76.788 |
| T120 | 79.37 | 73.83 |The values are expressed as means ± standard deviation of blood glucose and insulin at 30min, 60min, 90min, and 120min during OGTT. #, EX+VD, n=15; VD, n=13; EX, n=14; Con, n=13. *, P<0.05 compared with the values at the same time points using paired-t test.

## Slide 2
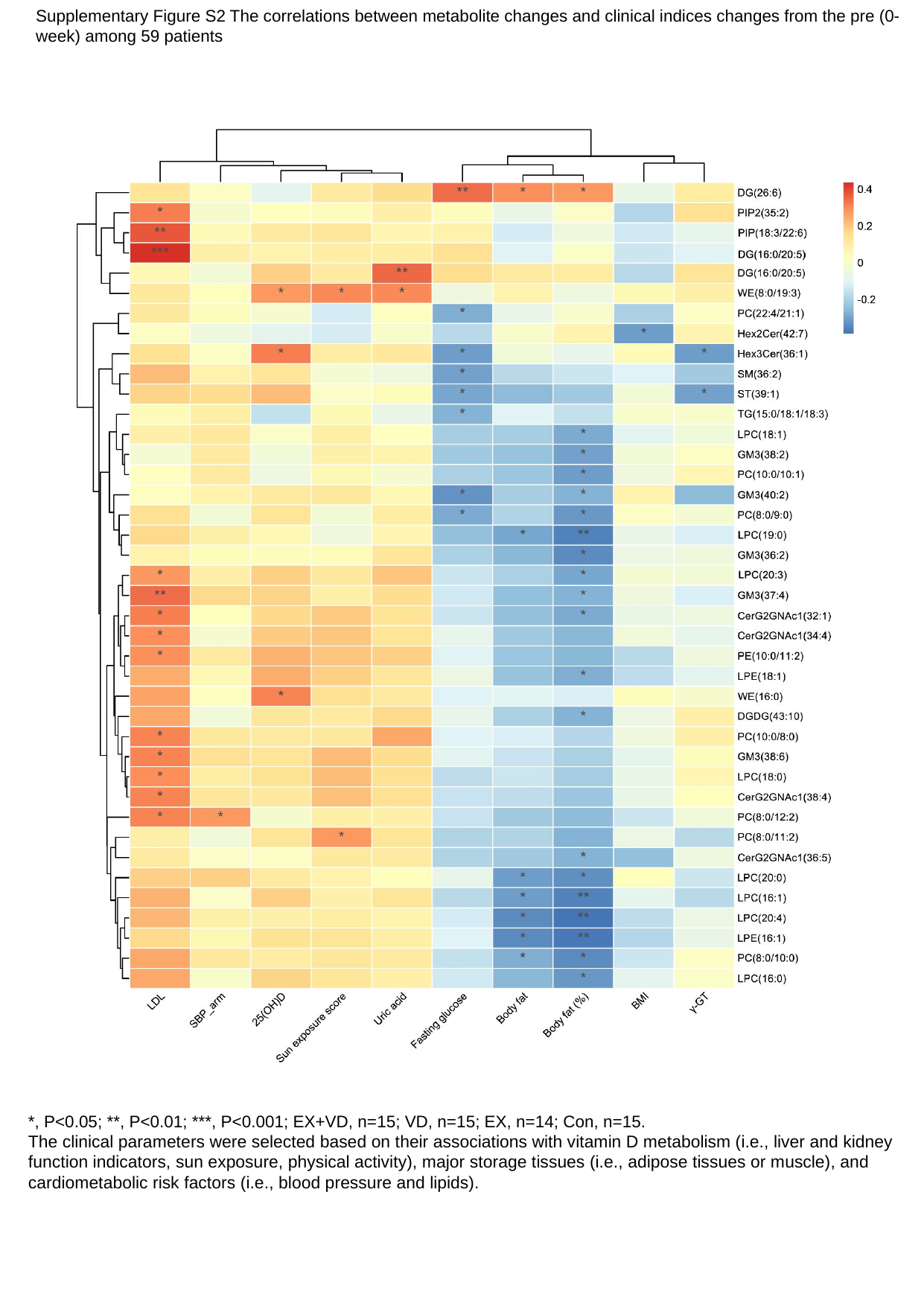

Supplementary Figure S2 The correlations between metabolite changes and clinical indices changes from the pre (0-week) among 59 patients
*, P<0.05; **, P<0.01; ***, P<0.001; EX+VD, n=15; VD, n=15; EX, n=14; Con, n=15.
The clinical parameters were selected based on their associations with vitamin D metabolism (i.e., liver and kidney function indicators, sun exposure, physical activity), major storage tissues (i.e., adipose tissues or muscle), and cardiometabolic risk factors (i.e., blood pressure and lipids).

## Slide 3
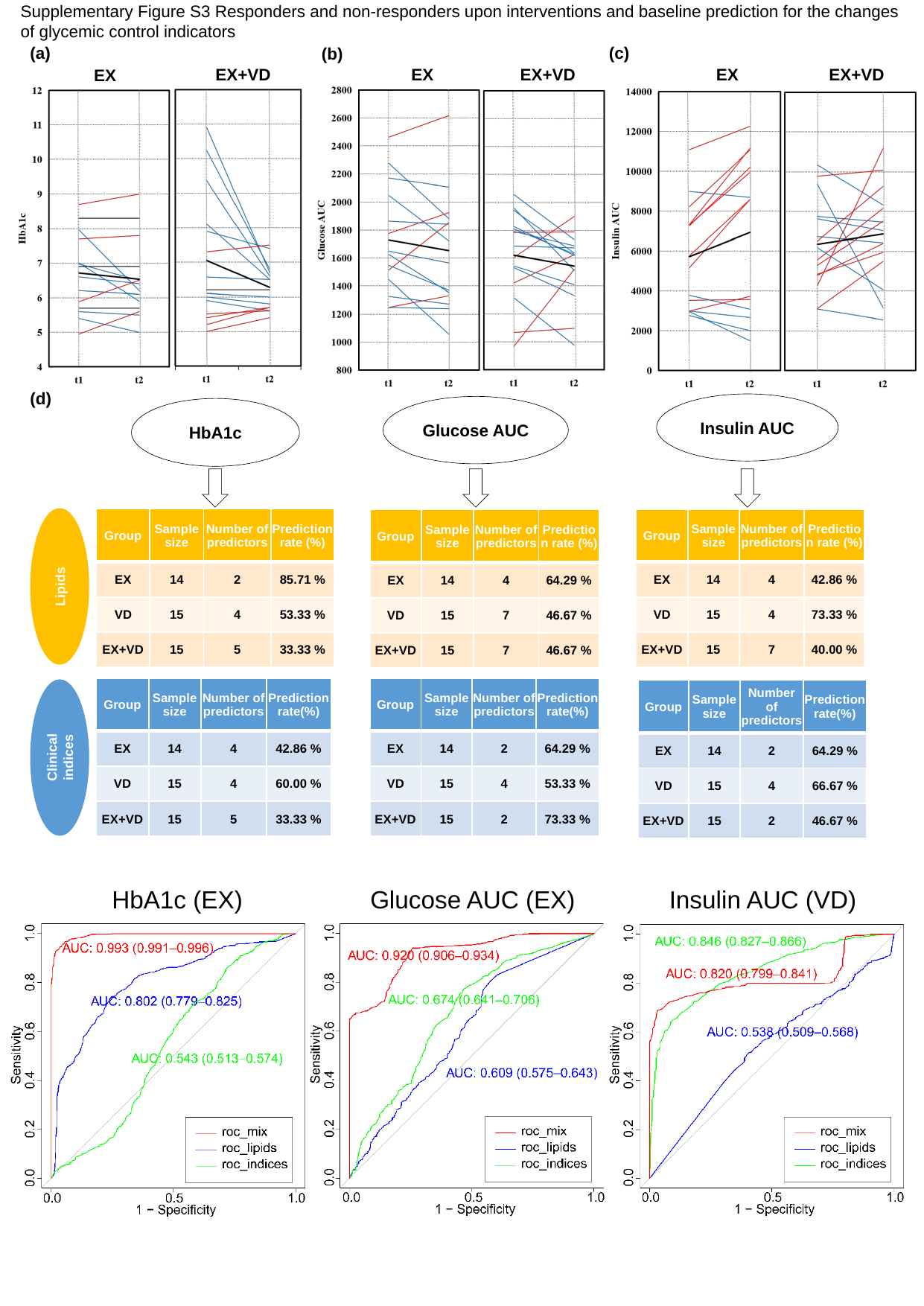

Supplementary Figure S3 Responders and non-responders upon interventions and baseline prediction for the changes of glycemic control indicators
(a)
(c)
(b)
EX+VD
EX
EX+VD
EX
EX+VD
EX
(d)
Insulin AUC
Glucose AUC
HbA1c
| Group | Sample size | Number of predictors | Prediction rate (%) |
| --- | --- | --- | --- |
| EX | 14 | 2 | 85.71 % |
| VD | 15 | 4 | 53.33 % |
| EX+VD | 15 | 5 | 33.33 % |
| Group | Sample size | Number of predictors | Prediction rate (%) |
| --- | --- | --- | --- |
| EX | 14 | 4 | 42.86 % |
| VD | 15 | 4 | 73.33 % |
| EX+VD | 15 | 7 | 40.00 % |
| Group | Sample size | Number of predictors | Prediction rate (%) |
| --- | --- | --- | --- |
| EX | 14 | 4 | 64.29 % |
| VD | 15 | 7 | 46.67 % |
| EX+VD | 15 | 7 | 46.67 % |
Lipids
| Group | Sample size | Number of predictors | Prediction rate(%) |
| --- | --- | --- | --- |
| EX | 14 | 4 | 42.86 % |
| VD | 15 | 4 | 60.00 % |
| EX+VD | 15 | 5 | 33.33 % |
| Group | Sample size | Number of predictors | Prediction rate(%) |
| --- | --- | --- | --- |
| EX | 14 | 2 | 64.29 % |
| VD | 15 | 4 | 53.33 % |
| EX+VD | 15 | 2 | 73.33 % |
| Group | Sample size | Number of predictors | Prediction rate(%) |
| --- | --- | --- | --- |
| EX | 14 | 2 | 64.29 % |
| VD | 15 | 4 | 66.67 % |
| EX+VD | 15 | 2 | 46.67 % |
Clinical indices
HbA1c (EX)
Glucose AUC (EX)
Insulin AUC (VD)

## Slide 4
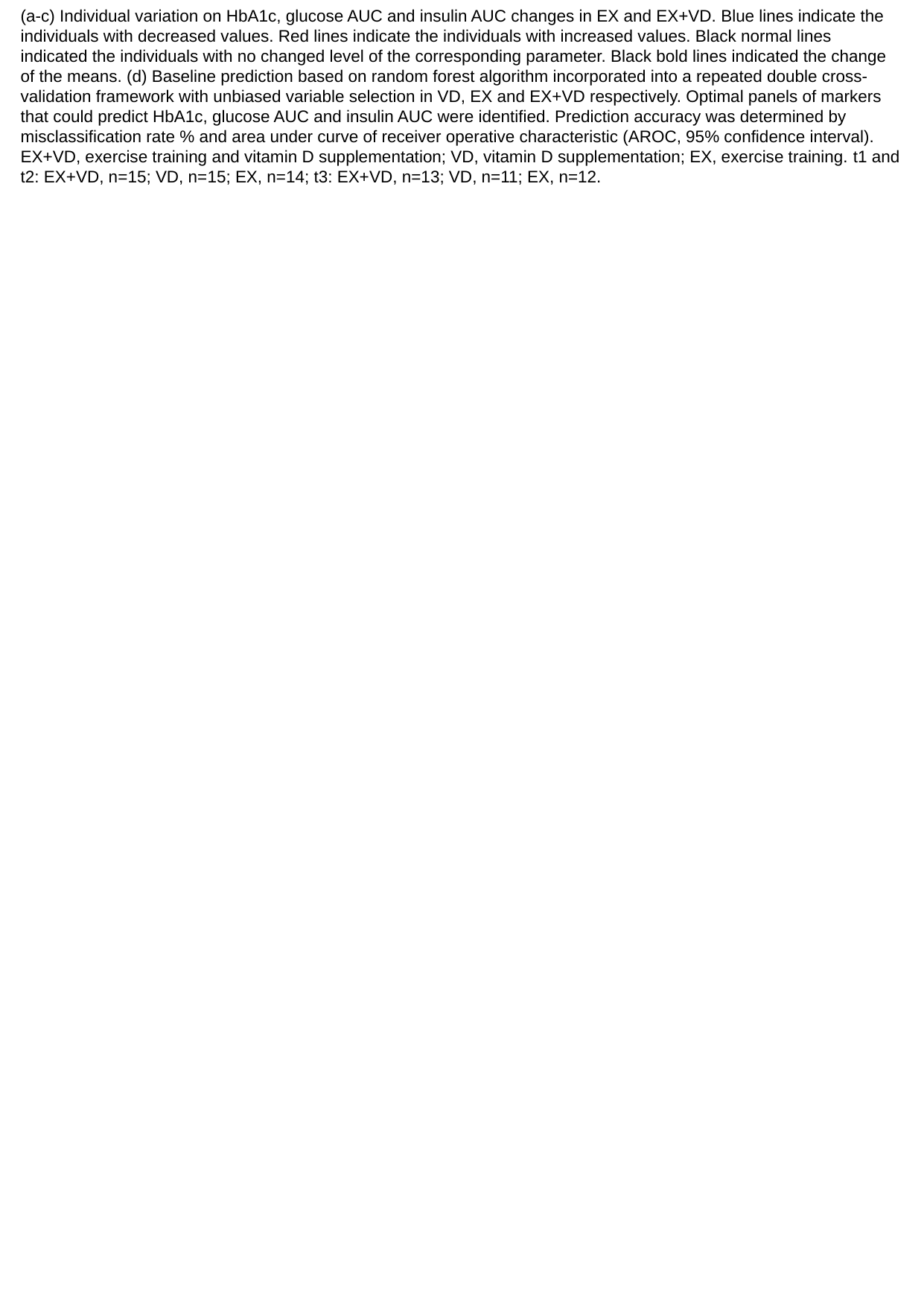

(a-c) Individual variation on HbA1c, glucose AUC and insulin AUC changes in EX and EX+VD. Blue lines indicate the individuals with decreased values. Red lines indicate the individuals with increased values. Black normal lines indicated the individuals with no changed level of the corresponding parameter. Black bold lines indicated the change of the means. (d) Baseline prediction based on random forest algorithm incorporated into a repeated double cross-validation framework with unbiased variable selection in VD, EX and EX+VD respectively. Optimal panels of markers that could predict HbA1c, glucose AUC and insulin AUC were identified. Prediction accuracy was determined by misclassification rate % and area under curve of receiver operative characteristic (AROC, 95% confidence interval). EX+VD, exercise training and vitamin D supplementation; VD, vitamin D supplementation; EX, exercise training. t1 and t2: EX+VD, n=15; VD, n=15; EX, n=14; t3: EX+VD, n=13; VD, n=11; EX, n=12.

## Slide 5
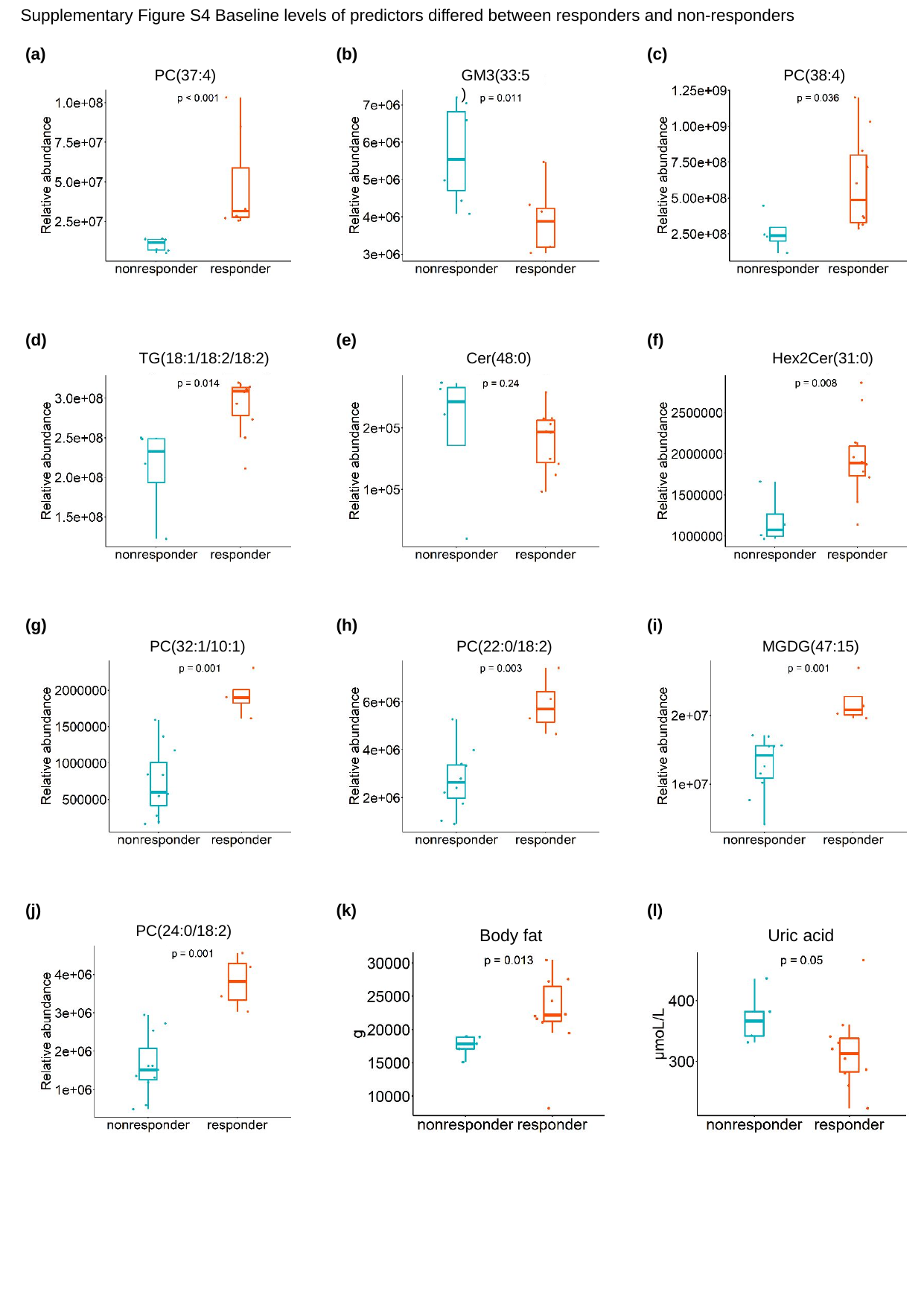

Supplementary Figure S4 Baseline levels of predictors differed between responders and non-responders
(a)
(b)
(c)
PC(37:4)
GM3(33:5)
PC(38:4)
(d)
(e)
(f)
TG(18:1/18:2/18:2)
Cer(48:0)
Hex2Cer(31:0)
Cer(d48:0)
(g)
(h)
(i)
PC(32:1/10:1)
PC(22:0/18:2)
MGDG(47:15)
(k)
(l)
(j)
PC(24:0/18:2)
Body fat
Uric acid

## Slide 6
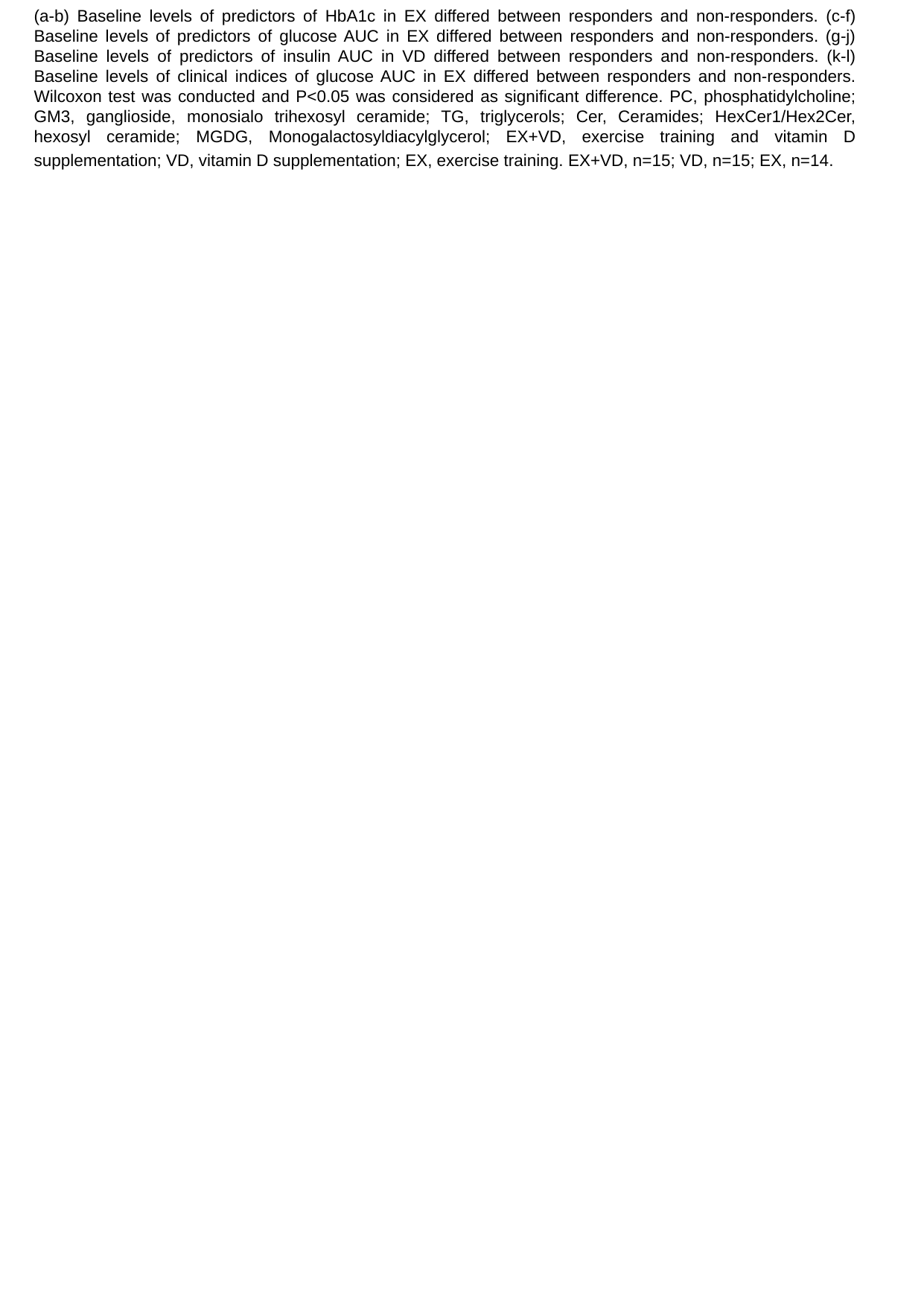

(a-b) Baseline levels of predictors of HbA1c in EX differed between responders and non-responders. (c-f) Baseline levels of predictors of glucose AUC in EX differed between responders and non-responders. (g-j) Baseline levels of predictors of insulin AUC in VD differed between responders and non-responders. (k-l) Baseline levels of clinical indices of glucose AUC in EX differed between responders and non-responders. Wilcoxon test was conducted and P<0.05 was considered as significant difference. PC, phosphatidylcholine; GM3, ganglioside, monosialo trihexosyl ceramide; TG, triglycerols; Cer, Ceramides; HexCer1/Hex2Cer, hexosyl ceramide; MGDG, Monogalactosyldiacylglycerol; EX+VD, exercise training and vitamin D supplementation; VD, vitamin D supplementation; EX, exercise training. EX+VD, n=15; VD, n=15; EX, n=14.
